# Revisão Sistemática sobre a Segurança do Exercício Físico na Cardiomiopatia Hipertrófica: Recomendações da Sociedade Brasileira de Cardiologia

**DOI:** 10.36660/abc.20260423

**Published:** 2026-09-08

**Authors:** Ricardo Stein, Rodrigo Otávio Bougleux Alô, Luiz Eduardo Fonteles Ritt, Alexsandro Alves Fagundes, Anderson Donelli da Silveira, Artur Haddad Herdy, Clea Colombo, Cristiano Faria Pisani, Fabrício Braga, Frank Nunes, Luciana Sacilotto, Marconi Gomes Silva, Mauricio Milani, Mauricio Pimentel, Milena dos Santos Barros Campos, Nágela Simão Vinhosa Nunes, Pablo Marino Nascimento, Susimeire Buglia, José Ronyeryson dos Santos Evangelista, Filipe Ferrari, Leonardo Castro Luna

**Affiliations:** 1 Hospital de Clínicas de Porto Alegre Porto Alegre RS Brasil Grupo de Pesquisa em Cardiologia do Exercício, Hospital de Clínicas de Porto Alegre, Porto Alegre, RS – Brasil; 2 Departamento de Clínica Médica Universidade Federal do Rio Grande do Sul Porto Alegre RS Brasil Departamento de Clínica Médica, Universidade Federal do Rio Grande do Sul, Porto Alegre, RS – Brasil; 3 Programa de Pós-Graduação em Cardiologia e Ciências Cardiovasculares Universidade Federal do Rio Grande do Sul Porto Alegre RS Brasil Programa de Pós-Graduação em Cardiologia e Ciências Cardiovasculares, Universidade Federal do Rio Grande do Sul, Porto Alegre, RS – Brasil; 4 Instituto Dante Pazzanese de Cardiologia São Paulo SP Brasil Instituto Dante Pazzanese de Cardiologia, São Paulo, SP – Brasil; 5 Hospital Cardio Pulmonar Salvador BA Brasil Hospital Cardio Pulmonar, Salvador, BA – Brasil; 6 Escola Bahiana de Medicina e Saúde Pública Pós-Graduação em Medicina e Saúde Humana Salvador BA Brasil Escola Bahiana de Medicina e Saúde Pública – Pós-Graduação em Medicina e Saúde Humana, Salvador, BA – Brasil; 7 Hospital Ana Nery Salvador BA Brasil Hospital Ana Nery, Salvador, BA – Brasil; 8 Hospital de Clínicas de Porto Alegre Porto Alegre RS Brasil Hospital de Clínicas de Porto Alegre, Porto Alegre, RS – Brasil; 9 Instituto de Cardiologia de Santa Catarina São José SC Brasil Instituto de Cardiologia de Santa Catarina, São José, SC – Brasil; 10 Faculdade São Leopoldo Mandic Campinas SP Brasil Faculdade São Leopoldo Mandic, Campinas, SP – Brasil; 11 Hospital das Clínicas Faculdade de Medicina Universidade de São Paulo São Paulo SP Brasil Instituto do Coração do Hospital das Clínicas da Faculdade de Medicina da Universidade de São Paulo, São Paulo, SP – Brasil; 12 Laboratório de Performance Humana Rio de Janeiro RJ Brasil Laboratório de Performance Humana, Rio de Janeiro, RJ – Brasil; 13 Universidade do Estado do Rio de Janeiro Rio de Janeiro RJ Brasil Universidade do Estado do Rio de Janeiro, Rio de Janeiro, RJ – Brasil; 14 Hospital César Leite Manhuaçu MG Brasil Hospital César Leite, Manhuaçu, MG – Brasil; 15 Universidade Federal de Minas Gerais Belo Horizonte MG Brasil Universidade Federal de Minas Gerais, Belo Horizonte, MG – Brasil; 16 Sportif - Clínica do Exercício e do Esporte Belo Horizonte MG Brasil Sportif - Clínica do Exercício e do Esporte, Belo Horizonte, MG – Brasil; 17 Fitcordis Brasília DF Brasil Fitcordis, Brasília, DF – Brasil; 18 Hospital São Lucas, Rede D’Or Aracaju SE Brasil Hospital São Lucas, Rede D’Or, Aracaju, SE – Brasil; 19 Complexo Hospitalar de Niterói Niterói RJ Brasil Complexo Hospitalar de Niterói, Niterói, RJ – Brasil; 20 Hospital Universitário Antônio Pedro Niterói RJ Brasil Hospital Universitário Antônio Pedro, Niterói, RJ – Brasil; 21 Instituto Nacional de Cardiologia Rio de Janeiro RJ Brasil Instituto Nacional de Cardiologia, Rio de Janeiro, RJ – Brasil; 22 Universidade do Grande Rio Rio de Janeiro RJ Brasil Universidade do Grande Rio, Rio de Janeiro, RJ – Brasil

**Keywords:** Cardiomiopatia Hipertrófica, Exercício Físico, Mortalidade, Revisão Sistemática, Arritmias Cardíacas

## Abstract

**Fundamento:**

Embora historicamente desaconselhado devido ao risco de arritmias e morte súbita cardíaca (MSC), o exercício físico (EF) pode ser seguro e benéfico para indivíduos com cardiomiopatia hipertrófica (CMH).

**Objetivo:**

Avaliar a associação entre a prática de EF e a incidência de mortalidade e de eventos arrítmicos em adultos portadores de CMH, estratificando a análise conforme os diferentes tipos e intensidades de exercício realizados.

**Métodos:**

Revisão sistemática de ensaios clínicos randomizados (ECRs) e estudos observacionais identificados nas bases de dados PubMed, Embase e Cochrane Library até dezembro de 2024. Foram incluídos estudos com indivíduos submetidos a diferentes intensidades de EF. O risco de viés foi avaliado pelas ferramentas RoB 2 e ROBINS-I, e a certeza da evidência, pela ferramenta GRADE. As recomendações foram elaboradas por um painel de especialistas da Sociedade Brasileira de Cardiologia.

**Resultados:**

Oito estudos (quatro ECRs e quatro observacionais; 9744 participantes) foram incluídos. Nenhum deles relatou aumento no risco de mortalidade ou de eventos arrítmicos graves associados ao EF, mesmo em intensidades moderadas ou vigorosas. A ocorrência de síncope foi rara. Além disso, um estudo observacional coreano, envolvendo mais de 7 mil participantes (idade média: 59 anos), demonstrou redução da mortalidade por todas as causas com maior intensidade de atividade física (curva dose-resposta). Em atletas de baixo risco, o esporte competitivo não se associou a eventos adversos graves. A qualidade da evidência variou de moderada a muito baixa.

**Conclusão:**

O EF na CMH deve ser incentivado com base em avaliação individualizada. Em pacientes sem critérios para alto risco de MSC, os benefícios parecem superar os riscos. Para subgrupos de maior risco, a recomendação é mais cautelosa e baseada em consenso de especialistas.

## Graphical abstract

**Figura Central:**
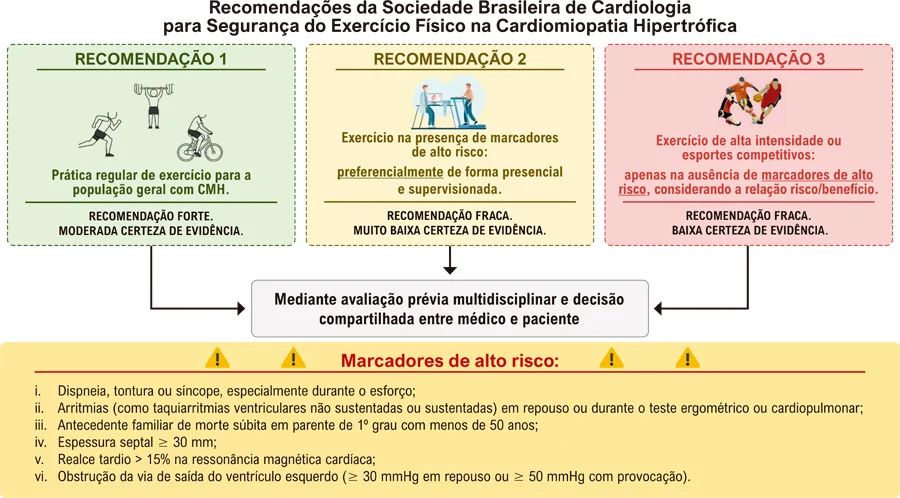
Revisão Sistemática sobre a Segurança do Exercício Físico na Cardiomiopatia Hipertrófica: Recomendações da Sociedade Brasileira de Cardiologia

**Recomendação 1:** A Sociedade Brasileira de Cardiologia recomenda a prática regular de exercício físico em pacientes com cardiomiopatia hipertrófica (CMH), ressaltando que a conduta terapêutica deve ser individualizada e estabelecida em decisão conjunta entre o médico e o paciente. Essa recomendação tem como objetivo incentivar pacientes com CMH a se tornarem fisicamente ativos, promovendo saúde global por meio da prática regular e progressiva de exercícios físicos, dentro de um contexto de prescrição individualizada.

## RECOMENDAÇÃO FORTE. MODERADA CERTEZA DA EVIDÊNCIA.

Essa recomendação baseia-se na ausência de aumento do risco de morte súbita associado à prática de exercício físico regular em indivíduos com CMH, conforme demonstrado em estudos clínicos que incluíram os seguintes subgrupos: pacientes sintomáticos (classe funcional da *New York Heart Association* [NYHA] II-III), portadores de cardioversor-desfibrilador implantável e pacientes submetidos a exercícios aeróbicos e/ou resistidos, realizados em diferentes intensidades.

A recomendação individual deve considerar o risco de morte súbita de cada paciente, estimado com base em características clínicas e por meio de uma calculadora de predição validada (HCM Risk-SCD 2023),^1^ com reavaliações periódicas anuais – ou semestrais, nos pacientes com marcadores de alto risco (ver abaixo) – e reavaliação imediata na presença de novos sintomas.

**Recomendação 2**: A Sociedade Brasileira de Cardiologia recomenda a prática regular de exercício físico em pacientes com CMH que apresentem marcadores de elevado risco para morte súbita, com vistas à melhoria da saúde global. Recomenda-se, preferencialmente, que o exercício seja realizado de forma supervisionada — como em programas de reabilitação cardiovascular ou em instalações equipadas com desfibrilador externo automático. Por fim, ressalta-se que a conduta terapêutica deve ser individualizada e definida por meio de decisão compartilhada entre o médico assistente e o paciente.

## RECOMENDAÇÃO FRACA. MUITO BAIXA CERTEZA DA EVIDÊNCIA.

São considerados marcadores de alto risco:

- sintomas como dispneia, síncope ou tontura, especialmente quando relacionados ao esforço;
- arritmias (como taquiarritmias ventriculares não sustentadas ou sustentadas) em repouso ou durante o esforço, bem como sintomas durante o teste ergométrico ou cardiopulmonar;
- antecedente familiar de morte súbita em parente de primeiro grau com menos de 50 anos;
- espessura septal igual ou superior a 30 mm;
- realce tardio extenso (> 15%) na ressonância magnética cardíaca;
- gradiente do trato de saída do ventrículo esquerdo ≥ 30 mmHg em repouso ou ≥ 50 mmHg com provocação, avaliado por ecocardiograma de exercício. A provocação pode ser realizada por manobra de Valsalva, esforço, pós-esforço ou após agachamento.

Essa abordagem deve incluir, idealmente, prescrição individualizada, monitoramento clínico, educação, suporte psicológico e controle de comorbidades, visando à segurança e à melhora da qualidade de vida. Deve-se considerar as preferências de cada indivíduo e, sempre que possível, associar exercícios aeróbicos e resistidos.

Essa recomendação baseia-se em consenso entre especialistas, uma vez que não há estudos específicos com o subgrupo de pacientes com marcadores de alto risco para morte súbita.

**Recomendação 3**: A Sociedade Brasileira de Cardiologia não contraindica a prática de exercícios de alta intensidade ou a participação em esportes competitivos por pacientes com CMH e, em casos criteriosamente selecionados, recomenda que tal possibilidade seja considerada. Para isso, é necessário que não estejam presentes marcadores de alto risco, que seja realizada avaliação prévia por equipe multidisciplinar e que a decisão final seja pactuada entre o médico assistente e o paciente (conforme critérios estabelecidos a seguir). Essa recomendação dirige-se especificamente a exercícios de alta intensidade e a atletas em contexto competitivo, nos quais o objetivo central costuma ser a performance e a competição.

## RECOMENDAÇÃO FRACA. BAIXA CERTEZA DA EVIDÊNCIA.

Essa recomendação baseia-se em poucos estudos observacionais e em consenso entre especialistas. Recomenda-se que os locais de treino e competição disponham de um plano de ação para assistência médica, com o objetivo de aumentar a segurança durante a prática desportiva.

A decisão deve considerar os seguintes critérios:

1. Ausência de marcadores de alto risco para eventos adversos ou morte súbita cardíaca (por exemplo: história de arritmias ventriculares, síncope inexplicada, espessura septal ventricular ≥ 30 mm ou realce tardio extenso na ressonância magnética cardíaca).
2. Avaliação prévia multidisciplinar, preferencialmente em centro de referência em cardiomiopatias hereditárias, incluindo:
  Teste de exercício (ergométrico ou ergoespirométrico) com análise da resposta pressórica;
  Monitorização eletrocardiográfica prolongada (por exemplo: Holter de 24 a 48 h);
  Ressonância magnética cardíaca com quantificação de fibrose miocárdica.
3. Estratificação individualizada do risco, preferencialmente pelo escore HCM Risk-SCD 2023,^1^seguida de decisão compartilhada com o paciente.

## Introdução

A cardiomiopatia hipertrófica (CMH) é caracterizada por hipertrofia do ventrículo esquerdo (VE) não explicada por sobrecarga hemodinâmica ou outras causas secundárias.^2,3^ O diagnóstico é definido por espessura da parede ventricular ≥ 15 mm ou, na presença de histórico familiar ou variante genética conhecida, ≥ 13 mm.^4^ A CMH apresenta expressiva heterogeneidade fenotípica, variando de formas assintomáticas a quadros graves, incluindo insuficiência cardíaca e morte súbita cardíaca (MSC).^5-7^Estudos populacionais, como o CARDIA, realizado nos Estados Unidos, estimaram uma prevalência de aproximadamente 0,2% (1:500) em adultos jovens assintomáticos,^8^enquanto análises genéticas mais recentes apontam prevalência próxima a 0,5% (1:200).^9,10^

Historicamente, o exercício físico (EF) tem sido desaconselhado em indivíduos com CMH devido ao risco percebido de arritmias e MSC, especialmente entre atletas jovens.^6,7^ No entanto, evidências recentes sugerem que, quando orientado com base em avaliação individualizada e estratificação de risco, o EF pode ser seguro e benéfico para essa população.^11-13^ Além disso, estudos indicam que o EF de alta intensidade pode ser bem tolerado e potencialmente vantajoso em pacientes criteriosamente selecionados.^12,13^ Ainda assim, persistem incertezas quanto à segurança de diferentes modalidades e intensidades de EF, especialmente em subgrupos de maior risco, o que reforça a necessidade de decisões individualizadas.

Com o objetivo de fundamentar a prática clínica nas melhores evidências científicas disponíveis, a Sociedade Brasileira de Cardiologia (SBC) elaborou a presente Recomendação Médica. Este documento analisa a relação entre a prática de EF e a ocorrência de eventos arrítmicos graves e mortalidade em pacientes com CMH, com análise estratificada de acordo com a intensidade do exercício e as características clínicas relevantes da doença.

## Métodos

Para fundamentar esta Recomendação da SBC, foi realizada uma revisão sistemática baseada em uma pergunta de pesquisa estruturada no formato PICO/PECO: “Em adultos com CMH, a prática de EF em diferentes tipos e intensidades está associada a maior risco de mortalidade, MSC ou arritmias ventriculares quando comparada à ausência de exercício ou à prática em intensidades mais leves?”.

O protocolo da revisão foi previamente registrado no *International Prospective Register of Systematic Reviews* (PROSPERO), sob o número CRD420251005992.

É importante frisar que a revisão sistemática rápida é uma modalidade desenvolvida para apoiar a tomada de decisão clínica a partir das melhores evidências disponíveis, mantendo o rigor científico, mas com adaptações que permitem maior agilidade na execução. Suas principais características incluem: restrição do idioma dos artigos ao inglês; busca de literatura cinzenta apenas nas referências dos estudos incluídos, com apoio de especialistas da área; e uso de inteligência artificial para auxiliar na triagem e extração dos dados. Essa estratégia tem sido amplamente adotada por sociedades médicas e instituições de saúde para a elaboração de recomendações clínicas.^14-16^

Uma descrição detalhada da metodologia empregada nesta revisão sistemática rápida pode ser encontrada no Material Suplementar.

### Critérios de elegibilidade

Foram incluídos estudos que apresentavam as seguintes características: (1) desenho observacional ou ensaios clínicos randomizados (ECRs); (2) inclusão de indivíduos com diagnóstico clínico ou genotípico de CMH, independentemente do sexo; (3) investigação da prática de EF, incluindo treinamento aeróbico, resistido, combinado (aeróbico + resistido) ou participação em modalidades esportivas específicas; (4) comparação entre indivíduos praticantes e não praticantes de EF, ou entre diferentes intensidades de EF; e (5) avaliação de pelo menos um dos seguintes desfechos: mortalidade por todas as causas, MSC e arritmias ventriculares (desfechos primários), além de arritmias supraventriculares, síncope, parada cardíaca abortada e choques adequados ou inadequados do cardioversor-desfibrilador implantável (CDI) (desfechos secundários).

Estudos que não atenderam a esses critérios ou que não avaliaram desfechos relevantes foram excluídos.

### Fontes de dados e estratégias de pesquisa

As buscas foram realizadas em três bases de dados: PubMed/MEDLINE, Embase e Cochrane Library, desde a data mais antiga disponível até 18 de dezembro de 2024. Adicionalmente, foi feita uma busca manual nas referências dos documentos recuperados e foram solicitados artigos de interesse aos especialistas da área.

### Seleção dos estudos

A seleção dos estudos, a extração das principais características e a avaliação da qualidade metodológica foram realizadas de forma independente por dois pesquisadores. Eventuais divergências foram resolvidas por consenso.

### Extração dos dados

Dois pesquisadores extraíram dados predefinidos em planilha eletrônica (Excel), incluindo informações de publicação, características clínicas e demográficas dos participantes e detalhes das intervenções (tipo de exercício, intensidade, frequência, duração e supervisão), além dos resultados dos desfechos de interesse.

### Avaliação da qualidade e risco de viés

Dois pesquisadores avaliaram, de forma independente, o risco de viés utilizando as ferramentas *Risk of Bias 2* (RoB 2)^17^ e *Risk Of Bias In Non-randomized Studies – of Interventions* (ROBINS-I).^18^ Divergências foram resolvidas por consenso. A certeza da evidência e a força das recomendações foram avaliadas de acordo com a ferramenta *Grading of Recommendations, Assessment, Development and Evaluations* (GRADE).^19^

### Análise dos dados

Para cada desfecho, foram extraídas as medidas de efeito reportadas nos estudos originais, incluindo risco relativo, razão de chances, *hazard ratio*, diferenças médias ou frequências absolutas, conforme aplicável. A elegibilidade dos estudos para cada síntese foi definida a partir da compatibilidade clínica e metodológica entre população, tipo/intensidade do exercício, comparadores e desfechos avaliados. Quando necessário, os dados foram reorganizados em tabelas padronizadas, com harmonização das informações disponíveis para facilitar a comparação entre os estudos.

Devido à heterogeneidade clínica e metodológica entre os desenhos dos estudos, intervenções, tempos de seguimento e definições dos desfechos, os resultados foram sintetizados de forma narrativa e suas características apresentadas em tabelas descritivas. Possíveis fontes de heterogeneidade foram exploradas qualitativamente, considerando desenho do estudo, intensidade do exercício, perfil de risco dos participantes e contexto da prática esportiva. A robustez das conclusões foi avaliada pela consistência dos achados entre estudos, interpretação do risco de viés e da precisão das estimativas.

### Recomendação clínica e conflitos de interesse

A recomendação clínica foi estabelecida de forma consensual durante reunião de um Painel de Recomendações da SBC, composto por especialistas cardiologistas atuantes e associados à sociedade. O projeto teve supervisão e financiamento da SBC, enquanto a elaboração da revisão sistemática e o processo de formulação das recomendações foram conduzidos por uma equipe independente de metodologistas (LCL, FF, JRSE). Essa equipe declarou não possuir conflitos de interesse relacionados à revisão, e os especialistas médicos do painel não receberam honorários nem apoio financeiro.

## Resultados

### Seleção dos estudos

Foram realizadas duas buscas independentes: uma para ECRs e outra para estudos observacionais, sendo identificadas 219 e 236 publicações, respectivamente. Após a remoção de duplicatas, oito estudos originais foram incluídos – quatro ECRs^11-13,20^e quatro estudos observacionais.^21-24^ Os fluxogramas de seleção (Figuras 1S e 2S), as estratégias de busca (Tabelas 1S e 2S) e a lista de estudos potencialmente elegíveis excluídos, com as respectivas justificativas (Tabela 3S), estão disponíveis no Material Suplementar.

### Características dos estudos

Os oito estudos incluídos abrangeram 9744 participantes, cujas características demográficas estão resumidas na Tabela 1 (dados detalhados na Tabela 4S). Os critérios de inclusão e exclusão dos estudos estão apresentados na Tabela 5S. O estudo observacional de Martinez et al.^24^não incluiu exclusivamente portadores de CMH; por isso, algumas variáveis demográficas e clínicas de interesse não estavam disponíveis separadamente para esses indivíduos.

**Tabela 1:** – Características dos estudos incluídos

Estudo	País	Desenho	Ano	Amostra (n)	Média de idade (anos)	Homens (%)	Espessura média máxima do VE (mm)
Basu et al.^13^	Reino Unido	ECR	2025	80	46	84	EF intenso: 16 Cuidados usuais: 16
Gudmundsdottir et al.^20^	Dinamarca	ECR	2024	59	58	73	EF moderado: 23 Cuidados usuais: 21
MacNamara et al.^12^	Estados Unidos	ECR	2023	15	48	67	EF moderado: 23 EF intenso: 24
Saberi et al.^11^	Estados Unidos	ECR	2017	136	50	58	EF moderado: 21 Cuidados usuais: 21
Kwon et al.^22^	Coreia do Sul	Coorte retrospectiva	2020	7666	60	70	NR
Lampert et al.^21^	Estados Unidos	Coorte prospectiva	2023	1660	39	60	Moderadamente ativos: 21,4 Vigorosamente ativos: 20 Sedentários: 21,9
Martinez et al.^24^	Estados Unidos	Coorte retrospectiva	2023	40	NR	NR	NR
Pelliccia et al.^23^	Itália	Coorte retrospectiva	2020	88	31 (mediana)	92	Atletas treinados: 17 Atletas destreinados: 17,3
**Estudo**	**CDI (%)**	**Obstrutivos (%)**		**FEVE (%)**	**Realce tardio**		**Sintomas**
Basu et al.^13^	EF intenso: 15 Cuidados usuais: 7,5	NR		65	NR		Participantes em classe funcional II da NYHA foram considerados elegíveis
Gudmundsdottir et al.^20^	EF moderado: 24 Cuidados usuais: 17	0		58,5	NR		Classe funcional II-III da NYHA: EF moderado: 86% Cuidados usuais: 70%
MacNamara et al.^12^	EF moderado: 63 EF intenso: 0	EF moderado: 25^≠^ EF intenso: 38^≠^		65,5	NR		NR
Saberi et al.^11^	EF moderado: 37,3 Cuidados usuais: 30,4	EF moderado: 16,4^¥^ Cuidados usuais: 17,4^¥^		70,7	% da massa do VE: EF moderado: 3,1 Cuidados usuais: 2,9		Classe funcional II-III da NYHA: EF moderado: 34,3% Cuidados usuais: 33,3%
Kwon et al.^22^	NR	NR			NR		NR
Lampert et al.^21^	Moderadamente ativos: 41,4 Vigorosamente ativos: 40,6 Sedentários: 49,8	Moderadamente ativos: 18,8^¥^ Vigorosamente ativos:11,9^¥^ Sedentários: 21,8^¥^		66	Leve/moderado (< 15%): Sedentários: 16,2% Moderadamente ativos: 18,3% Vigorosamente ativos: 17,6% Extenso (> 15%): Sedentários: 6,5% Moderadamente ativos: 6,2% Vigorosamente ativos: 6,9%		Classe funcional II da NYHA: 24% (fenótipo positivo)^†^
Martinez et al.^24^	NR	NR		NR	NR		NR
Pelliccia et al.^23^	2/88 (2,3)	NR		65	Atletas treinados: 22% Atletas destreinados: 39%		Atletas treinados: 22%^*^ Atletas destreinados: 25%^*^

CDI: cardioversor-desfibrilador implantável; ECR: ensaio clínico randomizado; EF: exercício físico; FEVE: fração de ejeção do ventrículo esquerdo; NR: Não reportado; NYHA: New York Heart Association; VE: ventrículo esquerdo. ≠ Obstrução provocável da via de saída do VE. ^¥^ Obstrução em repouso da via de saída do VE. ^†^ Refere-se aos participantes com cardiomiopatia hipertrófica fenotípica (n = 1534), dos quais 24% apresentavam dispneia aos esforços (classe funcional II da NYHA). * Valores calculados a partir da proporção de participantes assintomáticos (100% – % assintomáticos).

É importante destacar que subgrupos de potencial maior risco, como portadores de CDI, indivíduos com obstrução na via de saída do VE, pacientes com realce tardio (fibrose miocárdica) e aqueles sintomáticos, também estavam representados entre os indivíduos incluídos nos ECRs que avaliaram exercícios de intensidade moderada ou alta.

### Características das intervenções

As características das intervenções estão resumidas na Tabela 2 (dados detalhados na Tabela 6S). Nos quatro ECRs,^11-13,20^ as intervenções tiveram duração de 3^13,20^a 5 meses,^12^ incluindo treinamento contínuo moderado, intervalado (moderado ou intenso) e, em alguns casos, fortalecimento muscular. As sessões variaram de 20 a 60 minutos, com frequência de 3 a 7 vezes por semana e intensidade entre 60% e 95% da frequência cardíaca máxima ou de reserva. Um estudo foi totalmente presencial e supervisionado,^20^ outros combinaram sessões presenciais e domiciliares,^12,13^ e outro adotou treinos exclusivamente remotos.^11^

**Tabela 2:** – Intervenções dos estudos incluídos

Estudo	Intervenção	Intensidade	Volume	Frequência	Duração	Comparador	Principais resultados
Basu et al.^13^^¥^	EF intenso	70% da FCr, aumentando gradualmente até 85%	2 sessões de 1 h/semana + 1 h de EF domiciliar semanal	3x/semana	12 semanas	Cuidados usuais	Não houve diferença significativa no desfecho composto cardiovascular^†^ entre os grupos
Gudmundsdottir et al.^20^^¥^	EF moderado	60% da capacidade máxima de trabalho (escala de Borg 12-14)	10 min de aquecimento, 30 min de treino intervalado, 20 min de fortalecimento muscular	3x/semana	12 semanas	Cuidados usuais	Nenhum evento adverso maior^#^ foi observado em ambos os grupos
MacNamara et al.^12^^¥^	EF moderado e EF moderado + intenso progressivo	Moderado: 1-20 bpm abaixo do limiar ventilatório Intenso: 4×4 min a 90-95% da FCpico, com 3 min a ~70%	Moderado: até 4 sessões/semana (1×60 min + 3×30 min) + 1 sessão leve de recuperação Intenso: 14-15 sessões em 3 meses: 10 min de aquecimento + 4×4 min intervalado + 3 min de pausa ativa	~4x/semana	5 meses	Sem grupo controle sem exercício	Nenhuma arritmia grave ou evento cardíaco adverso ocorreu
Saberi et al.^11^	EF moderado	60% da FCr (Borg 11-14); progressão para 70% da FCr no 1º mês	20 min inicialmente; progressão até 60 min	Inicial: ≥ 3x/semana Progressão: 4-7x/semana	16 semanas	Cuidados usuais	Não houve eventos adversos maiores^⁋^ em nenhum dos grupos
Kwon et al.^22^	Participantes com o menor, intermediário ou maior nível de AF	Classificada em tercis do escore de AF*	Classificado em tercis do GEME, calculado considerando AFs com 3, 5 e 8 METs para intensidades leves, moderadas e vigorosas, respectivamente	NR	5,3 anos	AF de baixa intensidade	AF moderada a vigorosa associou-se a menor mortalidade geral e tendência a menor mortalidade cardiovascular
Lampert et al.^21^	EF moderado ou vigoroso	Moderado: 4,0-5,9 METs Vigoroso: ≥ 6,0 METs	Moderado: ≥ 60 h/ano em atividades de 4 a < 6 METs Vigoroso: ≥ 60 h/ano em atividades ≥ 6 METs	NR	38 meses	Sedentário	EF vigoroso não aumentou mortes ou arritmias graves em comparação ao EF moderado ou sedentarismo
Martinez et al.^24^	Retorno ao esporte	NR (presumivelmente alta, dado o nível competitivo)	NR (presumivelmente alta, dado o nível competitivo)	NR	7 anos	-	O retorno à prática esportiva é viável e está associado a baixas taxas de eventos adversos, mesmo em atletas de elite
Pelliccia et al.^23^	Manutenção do treinamento de alta intensidade	Treinamento de alta intensidade	Grupo treinado: ≥ 3 sessões/semana, ≥ 2 h por sessão, total 6-14 h/semana Grupo destreinado: < 6 h/semana (atividade sedentária ou ocasional)	≥ 3x/semana	7 anos	Sedentário ou atividade ocasional de lazer < 6 h/semana	Em atletas de baixo risco, manter a competição não aumentou eventos cardíacos nem causou piora clínica

AF: atividade física; EF: exercício físico; FC: frequência cardíaca; FCr: frequência cardíaca de reserva; GEME: gasto energético mínimo estimado, calculado em MET-min/semana com base na frequência semanal e na duração mínima das atividades físicas (20 min para vigorosas e 30 min para moderadas e leves); METs: equivalentes metabólicos; NR: Não reportado. *Calculado como média diária ponderada em METs/dia: (8a + 5b + 3c)/7, onde a, b e c representam os dias com atividade vigorosa, moderada e leve, respectivamente. ^¥^Incluiu intervenções supervisionadas. ^#^Morte, parada cardíaca abortada, taquicardia ventricular sustentada, choque de cardioversor-desfibrilador implantável ou internações. ^⁋^Morte, parada cardíaca abortada, choques apropriados de cardioversor-desfibrilador implantável ou taquicardia ventricular sustentada. ^†^Morte, parada cardíaca, terapia apropriada/inapropriada com cardioversor-desfibrilador implantável, síncope induzida por EF, taquicardia ventricular sustentada, taquicardia ventricular não sustentada, arritmias atriais sustentadas ≥ 30 s.

Entre os estudos observacionais, dois estratificaram indivíduos da população geral de CMH em três níveis diferentes de atividade física baseado em equivalentes metabólicos (METs).^21,22^ Os outros dois estudos observacionais se concentraram na avaliação de atletas portadores de CMH: Pelliccia et al.^23^ avaliaram atletas que mantiveram ou interromperam treinos de alta intensidade estruturados (≥ 6 h/semana), enquanto Martinez et al.^24^ acompanharam atletas semiprofissionais e profissionais que retornaram ou não ao exercício intenso após decisão compartilhada.

Informações sobre registro, suporte financeiro e conflitos de interesse dos estudos incluídos estão apresentados na Tabela 7S. O checklist PRISMA deste documento encontra-se na Tabela 8S.

### Desfechos avaliados

#### Mortalidade

Nenhum dos ECRs relatou mortes relacionadas aos programas de EF, independentemente da intensidade.^11-13,20^ De forma semelhante, os estudos observacionais não identificaram aumento no risco de mortalidade associado à prática regular de EF.^21-24^

Em um estudo observacional com 7666 indivíduos (idade média de 59 anos), a mortalidade total e cardiovascular diminuiu progressivamente com níveis cada vez mais elevados de atividade física autorreferida (curva dose-resposta).^22^Em outro estudo, realizado com indivíduos mais jovens (idade média de 39 anos), o EF vigoroso não aumentou o risco de morte ou arritmias graves em relação aos grupos classificados como EF moderado ou sedentário no estudo.^21^

Nas duas coortes compostas exclusivamente por atletas com CMH,^23,24^ manter-se em competição por até sete anos não se associou a maior risco de morte súbita ou piora clínica, e os eventos clínicos observados não tiveram relação com a prática de EF.

#### Arritmias, choques de CDI e síncopes

De modo geral, os estudos não evidenciaram (quando reportado) aumento de arritmias ventriculares sustentadas, choques de CDI ou síncopes associadas ao EF. Episódios de taquicardia ventricular não sustentada foram relatados em ambos os grupos (intervenção e controle), sem diferenças significativas.^12,13^ Um único caso de taquicardia ventricular sustentada, sem complicações, ocorreu no grupo controle de um ECR.^13^Martinez et al.^24^ relataram, entre atletas portadores de CMH que retornaram ao esporte, um evento isolado de parada cardíaca não relacionada ao esforço e um caso de síncope não arrítmica por esforço, com retorno ao esporte e ausência de novos episódios por 52 meses.^24^

Informações detalhadas sobre esses desfechos em cada estudo estão apresentadas na Tabela 9S.

#### Risco de viés

De acordo com a ferramenta RoB 2, dois ECRs foram classificados como de alto risco de viés, um, como de risco moderado e um, como de baixo risco (Figura 1). Entre os estudos observacionais avaliados pelo ROBINS-I, dois apresentaram alto risco de viés; um, risco moderado, e um, risco baixo (Figura 2).

**Figura 1:**
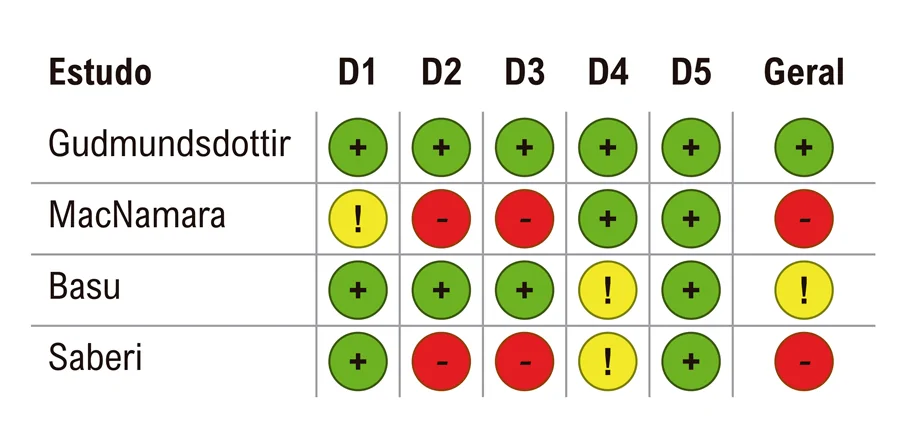
– RoB 2 dos ensaios clínicos randomizados. D1: Randomização; D2: Desvios no tratamento; D3: Dados faltantes; D4: Medida do desfecho; D5: Seleção de resultados reportados.

**Figura 2:**
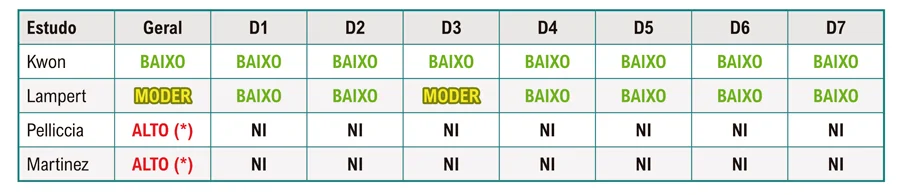
– ROBINS-I dos estudos observacionais. (*) Não houve controle de confundidores - Alto risco de viés; NI: Não informado. D1: Confundimento; D2: Classificação das intervenções; D3: Seleção dos participantes; D4: Desvio das intervenções; D5: Dados faltantes; D6: Medida dos desfechos; D7: Viés de relato.

## Discussão

Esta revisão sistemática fundamenta as recomendações da SBC sobre a segurança do EF em indivíduos com CMH. Com base nos achados, discutem-se os potenciais riscos do EF em três grupos: indivíduos com CMH da população geral, aqueles com marcadores de alto risco para MSC e atletas semiprofissionais ou profissionais.

### Contexto histórico e atual

Até um passado recente, as diretrizes desencorajavam a prática de EF em indivíduos com CMH, principalmente pelo temor de MSC em atletas jovens. As Conferências de Bethesda,^25,26^ a Sociedade Europeia de Cardiologia (2005)^27^ e a *American Heart Association/American College of Cardiology* (2015)^28^ adotavam uma postura restritiva em relação à prática de esportes recreativos ou competitivos de intensidade moderada a vigorosa em pessoas com CMH.

Essa restrição pode contribuir para o aumento do comportamento sedentário (caracterizado por atividades realizadas em vigília com gasto energético ≤ 1,5 METs, geralmente em posição sentada, reclinada ou deitada), da inatividade física (não cumprimento das recomendações mínimas de atividade física para a saúde), da obesidade, da ansiedade e também para a piora da qualidade de vida.^29,30^ A resistência à prescrição de EF neste contexto persiste até os dias atuais, em parte pelo impacto emocional de casos de MSC e também pelo desconhecimento de evidências atuais. Entretanto, diretrizes mais recentes da *American Heart Association/American College of Cardiology* (2025)^31^ indicam que atletas com CMH, inclusive portadores de variantes genéticas patogênicas e atletas de elite, podem ser elegíveis para participar de exercícios vigorosos e esportes competitivos. Essa liberação, contudo, está condicionada a uma avaliação especializada e a uma decisão compartilhada. Os dados disponíveis indicam que não há aumento significativo no risco de eventos cardíacos graves.

### Consequências da restrição ao exercício e da inatividade física em pacientes com cardiomiopatia hipertrófica

Em pacientes com CMH, particularmente quando há contraindicação formal ao EF, podem ser perdidos os benefícios associados à prática regular de exercício sobre a saúde cardiovascular, metabólica, musculoesquelética e mental. Além disso, a redução ou interrupção da prática de EF pode favorecer o desenvolvimento ou agravamento de alterações metabólicas e aumentar o risco cardiovascular individual, de forma semelhante ao observado na população geral.^32-38^

Estudo qualitativo externo a esta revisão sistemática sugere que adultos ativos ou atletas com CMH podem apresentar dificuldade de adaptação às restrições esportivas, especialmente quando há histórico competitivo, forte identidade atlética ou redução expressiva do tempo dedicado ao exercício.^39^ Considerando suas limitações metodológicas, esses dados podem ser utilizados para contextualizar valores e preferências dos pacientes. Portanto, apesar de não fundamentarem recomendações clínicas rígidas, esses aspectos reforçam a importância de o cardiologista considerar aspectos psicossociais, oferecer suporte emocional adequado e incorporar as preferências do paciente durante o processo de decisão compartilhada.

### Evidências dos ensaios clínicos randomizados

Apesar de limitações, como seguimento curto, amostras pequenas e predominância de estudos conduzidos em países de alta renda,^11-13,20^os ECRs disponíveis não registraram eventos adversos significativos, mesmo em protocolos de alta intensidade. No total, os estudos acompanharam aproximadamente 80 pacientes-ano sem ocorrência de eventos adversos relevantes, sugerindo que o EF intenso pode ser seguro em pacientes com CMH.

Todos os quatro ECRs também observaram melhora na capacidade funcional, independentemente da intensidade ou duração do protocolo. Em um ensaio multicêntrico com 136 pacientes, o consumo máximo de oxigênio de pico (VO_2_ pico) aumentou em média 1,27 mL^-1^.Kg.^-1^.min após 16 semanas de EF moderado,^11^ enquanto Basu et al.^13^ relataram um ganho médio de 4,1 mL^-1^.Kg.^-1^.min com EF de alta intensidade por 12 semanas. Parte desses ganhos, porém, foi perdida após seis meses, evidenciando a importância da continuidade do treinamento.

Estas características de segurança e benefícios cardiometabólicos contribuíram para as Recomendações 1 e 2.

### Evidências dos estudos observacionais

Nesta revisão, foram incluídos quatro estudos observacionais: dois em atletas com CMH^23,24^ e dois com indivíduos da população geral portadores da doença.^21,22^ Embora estudos observacionais apresentem, em geral, baixa qualidade de evidência (GRADE), eles são úteis para avaliar a segurança do EF em seguimentos prolongados e seu impacto sobre a mortalidade, devido às amostras mais robustas.

Nos estudos com atletas, o retorno ao esporte de alto rendimento, após avaliação especializada e decisão compartilhada, foi considerado seguro. Martinez et al.^24^ registraram baixa incidência de eventos em 40 atletas com CMH acompanhados por uma equipe multidisciplinar, sem ocorrência de mortes durante um seguimento médio de sete anos. Em outro estudo retrospectivo, 27 dos 88 atletas com CMH mantiveram os treinos sem eventos adversos e com estabilidade clínica.^23^Esses achados indicam que, em atletas bem avaliados, a prática esportiva de alto rendimento pode ser viável e segura. Entretanto, a força das recomendações é limitada pela natureza retrospectiva e tamanho amostral modesto. Esses achados foram incorporados na Recomendação 3 deste documento.

Nos estudos realizados na população geral com CMH, os resultados também foram favoráveis. Em uma coorte com mais de 7000 indivíduos (média de idade de 59 anos), observou-se redução progressiva da mortalidade com o aumento da intensidade da atividade física (curva dose-resposta).^22^ Por esse estudo ter sido avaliado como de baixo risco de viés, esse dado resultou na elevação da certeza da evidência de “Baixa” para “Moderada” quanto ao benefício do exercício e o desfecho mortalidade.

Consistentemente, outro estudo com mais de 1600 participantes (idade média de 39 anos) demonstrou que a prática de EF vigoroso – inclusive em atletas de nível competitivo (259 participantes) – não foi associada a um risco aumentado de eventos adversos quando comparada à prática de EF de menor intensidade.^21^Esses dados de segurança e mortalidade contribuíram para as Recomendações 1 e 2.

### Evidências de estudos observacionais não incluídos nesta revisão

Dois estudos observacionais, não incluídos nesta revisão por critérios de elegibilidade, mas que fornecem informações relevantes sobre o tema, são descritos a seguir.

Basu et al.^40^ realizaram um estudo com 53 atletas – todos com diagnóstico de CMH descrita como morfologicamente leve e de baixo risco para MSC. Durante um seguimento de 4,5 anos, não foram identificados casos de morte súbita, taquicardia ventricular sustentada ou síncope associados à manutenção da prática esportiva competitiva. A taxa de arritmias assintomáticas descrita foi de 2,1% ao ano, com impacto clínico mínimo.^40^

Em outro estudo observacional, com seguimento de quase nove anos, Desai et al.^41^ descreveram que indivíduos portadores de CMH assintomáticos ou minimamente sintomáticos que atingiram > 100% dos METs previstos no teste de exercício apresentaram taxas muito baixas de eventos, enquanto aqueles com menor capacidade funcional (< 85%) tiveram risco significativamente maior.^41^ Esse achado reforça a relevância da capacidade funcional não apenas como desfecho de eficácia, mas também como marcador prognóstico e ferramenta para estratificação de risco.

### Subgrupos com características específicas

É digno de nota que indivíduos portadores de CDI, pacientes sintomáticos (classe funcional II-III da NYHA), aqueles com obstrução da via de saída do VE (em repouso ou provocável) e pacientes com fibrose miocárdica dita leve (< 15%) foram incluídos nos estudos. O realce tardio extenso (> 15%) esteve presente em 6 a 7% dos participantes em um dos estudos.^21^ Portanto, a recomendação favorável ao EF aplica-se à maioria dos pacientes de baixo risco, inclusive àqueles que apresentem essas características, devendo-se individualizar a abordagem nos casos de maior risco.

### Realce tardio na ressonância magnética cardíaca

O realce tardio é um marcador prognóstico relevante na CMH, associado a maior risco de arritmias ventriculares e MSC, sobretudo quando excede 15% da massa do VE – valor adotado neste documento como critério de alto risco para MSC.

Embora não haja consenso sobre a melhor técnica de quantificação, a presença de realce tardio pode auxiliar na estratificação de risco, especialmente em casos de indicação ambígua de CDI, e na diferenciação entre CMH e outras causas de hipertrofia ventricular.^42,43^Entretanto, a relação direta entre realce tardio e prática de EF ainda não foi estabelecida por estudos específicos e conclusivos.

### Pacientes com marcadores de alto risco para morte súbita cardíaca

Um dos maiores desafios deste documento foi abordar pacientes com CMH e marcadores de alto risco para MSC. Não há evidências específicas sobre a segurança do EF nesse subgrupo. Assim, a recomendação da SBC, baseada em consenso e cautela, é favorável à prática de EF, desde que condicionada à avaliação individualizada e à tomada de decisão compartilhada, reconhecendo-se a muito baixa certeza das evidências disponíveis.

### Limitações e perspectivas futuras

Este documento apresenta limitações importantes. A primeira refere-se à dificuldade de conduzir estudos metodologicamente robustos e com seguimento prolongado em doenças de baixa prevalência, o que restringe a extrapolação dos resultados para a população geral com CMH.

A segunda limitação diz respeito à heterogeneidade das intervenções avaliadas, tanto em termos de modalidade e intensidade, quanto à diversidade clínica dos participantes. Essa variabilidade compromete a robustez das conclusões e exige interpretação cautelosa.

Os adultos jovens (20-40 anos), subgrupo tradicionalmente de maior risco para MSC, foram avaliados apenas em pequenos estudos observacionais, reduzindo a confiança das recomendações para esse perfil. Essa cautela é reforçada por dados epidemiológicos externos, no qual as causas cardiovasculares foram a principal categoria de morte súbita em adultos jovens de 18-45 anos, incluindo alguns casos de CMH identificados em autópsia.^44^

Adicionalmente, a aplicabilidade ao contexto brasileiro pode ser limitada por fatores como acesso restrito a especialistas e escassez de serviços de reabilitação cardíaca. Além disso, no cenário desta Recomendação, desconhecemos estudos realizados com pacientes brasileiros diagnosticados com CMH.

Frente a essas limitações, são necessários novos estudos – incluindo registros prospectivos e ECRs com maior seguimento – para fortalecer a base de evidências e, futuramente, revisar as recomendações aqui apresentadas.

## Conclusão

O EF e a prática esportiva no cenário da CMH devem ser incentivados com base em avaliação individualizada, considerando fatores como perfil clínico, risco cardiovascular e preferências do paciente. Em pacientes sem critérios de alto risco para MSC, os benefícios do EF, incluindo melhora da capacidade funcional, qualidade de vida e possíveis efeitos cardioprotetores, parecem superar os riscos.

No entanto, para subgrupos de maior risco, como aqueles com arritmias graves, história de parada cardíaca ou disfunção ventricular significativa, a recomendação deve ser mais cautelosa. Nesses casos, a decisão deve ser tomada por meio de uma avaliação multidisciplinar e de um processo de decisão compartilhada com o paciente, após uma análise criteriosa da relação risco-benefício. Estudos adicionais são necessários para melhor estratificar os riscos e definir protocolos personalizados de EF nessa população.

## *Material suplementar

MATERIAL SUPLEMENTAR Para informação adicional, por favor, clique aqui https://abccardiol.org/supplementary-material/2026/12308/2026-0423_AO_Material-suplementar.pdf
