# Supplementary material for: Revisão Sistemática sobre a Segurança do Exercício Físico na Cardiomiopatia Hipertrófica: Recomendações da Sociedade Brasileira de Cardiologia
Source: Arq Bras Cardiol. 2026 Sep 8;123(8):e20260423. [Article in Portuguese] doi: 10.36660/abc.20260423 (PMC13671148; doi:10.36660/abc.20260423)

MATERIAL SUPLEMENTAR

Revisão sistemática sobre a segurança do exercício físico na cardiomiopatia hipertrófica: recomendações da Sociedade Brasileira de Cardiologia

Índice

Detalhes da metodologia da revisão sistemática rápida

Tabela 1S. Estratégia de busca por ensaios clínicos randomizados

Tabela 2S. Estratégia de busca por estudos observacionais

Tabela 3S. Lista de estudos potencialmente elegíveis excluídos

Tabela 4S. Características demográficas dos pacientes dos estudos incluídos

Tabela 5S. Critérios de inclusão e exclusão dos estudos incluídos

Tabela 6S. Características das intervenções dos estudos incluídos

Tabela 7S. Conflitos de interesse, DOIs, registro e suporte financeiro dos estudos

Tabela 8S. Checklist PRISMA da revisão sistemática

Tabela 9S. Eventos relacionados a arritmias, choques de CDI e síncopes dos estudos incluídos

Figura 1S. Fluxograma PRISMA da seleção de ensaios clínicos randomizados

Figura 2S. Fluxograma PRISMA da seleção de estudos observacionais

Detalhes da metodologia da revisão sistemática rápida

Com base na pergunta estruturada e de acordo com a estratégia PICO/PECO, foram realizadas buscas nas bases de dados MEDLINE (via PubMed), Embase e Cochrane Library. As buscas foram conduzidas em 18 de dezembro de 2024, com estratégias específicas para identificar ensaios clínicos randomizados e estudos observacionais, elaboradas a partir dos vocabulários controlados de cada base e com restrição de idioma ao inglês. Foram utilizados apenas descritores relacionados à condição (cardiomiopatia hipertrófica) e às intervenções (exercício físico e atividade física), com o objetivo de maximizar a sensibilidade da busca. Buscas de literatura cinzenta foram realizadas apenas nas referências dos estudos incluídos e com o apoio de especialistas da área.

Foram realizadas duas buscas independentes: uma para ensaios clínicos randomizados e outra para estudos observacionais, resultando na identificação de 219 e 236 publicações, respectivamente. A triagem dos estudos foi conduzida com o auxílio do *software* Rayyan por dois revisores independentes, sendo as divergências resolvidas por consenso, em duas etapas. Na primeira etapa, foram analisados os títulos e resumos para pré-seleção dos estudos potencialmente elegíveis. Na segunda etapa, procedeu-se à leitura na íntegra dos textos selecionados para confirmação da elegibilidade e inclusão final.

Após a remoção de duplicatas e a aplicação dos critérios de elegibilidade, foram incluídos oito estudos originais: quatro ensaios clínicos randomizados e quatro estudos observacionais, conforme ilustrado nos fluxogramas PRISMA (**Figuras 1S e 2S**).

A extração dos dados foi realizada por dois revisores, de forma independente, utilizando planilha previamente estruturada. Foram coletadas as seguintes informações: características dos estudos, dados clínicos e demográficos dos participantes, intervenções (tipo, intensidade, frequência, duração e supervisão do exercício físico) e desfechos avaliados. Os desfechos primários incluíram mortalidade por todas as causas, morte súbita cardíaca e arritmias ventriculares; os secundários englobaram arritmias supraventriculares, síncope, parada cardíaca abortada e choques de cardioversor-desfibrilador implantável (apropriados ou inapropriados). Divergências na extração foram resolvidas por consenso.

A qualidade metodológica foi avaliada por dois revisores independentes, utilizando as ferramentas RoB 2 (para ensaios clínicos randomizados) e ROBINS-I (para estudos observacionais). A qualidade da evidência e a força das recomendações foram classificadas de acordo com os critérios GRADE. Eventuais discordâncias foram resolvidas por consenso.

Tabela 1S. Estratégia de busca por ensaios clínicos randomizados.

**PubMed/MEDLINE**	((Cardiomyopathy, Hypertrophic[MeSH Terms]) OR (Hypertrophic Cardiomyopathy[Title/Abstract]) OR (Hypertrophic Cardiomyopathies[Title/Abstract]) OR (Cardiomyopathies, Hypertrophic[Title/Abstract]) OR (Cardiomyopathy, Hypertrophic Obstructive[Title/Abstract]) OR (Cardiomyopathies, Hypertrophic Obstructive[Title/Abstract]) OR (Hypertrophic Obstructive Cardiomyopathies[Title/Abstract]) OR (Hypertrophic Obstructive Cardiomyopathy[Title/Abstract]) OR (Obstructive Cardiomyopathies, Hypertrophic[Title/Abstract]) OR (Obstructive Cardiomyopathy, Hypertrophic[Title/Abstract]) OR (Non-obstructive Hypertrophic Cardiomyopathy[Title/Abstract]) OR (Hypertrophic Non-obstructive Cardiomyopathy[Title/Abstract]) OR (Cardiomyopathy, Hypertrophic Non-obstructive[Title/Abstract]))<br><br>AND<br><br>((Exercise[MeSH Terms]) OR (Exercises[Title/Abstract]) OR (Exercise, Physical[Title/Abstract]) OR (Exercises, Physical[Title/Abstract]) OR (Physical Exercise[Title/Abstract]) OR (Physical Exercises[Title/Abstract]) OR (Physical Activity[Tit((Exercise[MeSH Terms]) OR (Exercises[Title/Abstract]) OR (Exercise, Physical[Title/Abstract]) OR (Exercises, Physical[Title/Abstract]) OR (Physical Exercise[Title/Abstract]) OR (Physical Exercises[Title/Abstract]) OR (Physical Activity[Title/Abstract]) OR (Activities, Physical[Title/Abstract]) OR (Activity, Physical[Title/Abstract]) OR (Physical Activities[Title/Abstract]) OR (Exercise, Aerobic[Title/Abstract]) OR (Aerobic Exercise[Title/Abstract]) OR (Aerobic Exercises[Title/Abstract]) OR (Exercises, Aerobic[Title/Abstract]) OR (Exercise, Isometric[Title/Abstract]) OR (Exercises, Isometric[Title/Abstract]) OR (Isometric Exercises[Title/Abstract]) OR (Isometric Exercise[Title/Abstract]) OR (Acute Exercise[Title/Abstract]) OR (Acute Exercises[Title/Abstract]) OR (Exercise, Acute[Title/Abstract]) OR (Exercises, Acute[Title/Abstract]) OR (Exercise Training[Title/Abstract]) OR (Exercise Trainings[Title/Abstract]) OR (Training, Exercise[Title/Abstract]) OR (Trainings, Exercise[Title/Abstract]) OR (Sports[MeSH Terms]) OR (Sport[Title/Abstract]) OR (Athletics[Title/Abstract]) OR (Athletic[Title/Abstract]) OR (Team Sports[MeSH Terms]) OR (Team Sport[Title/Abstract]) OR (Resistance Training[MeSH Terms]) OR (Training, Resistance[Title/Abstract]) OR (Strength Training[Title/Abstract]) OR (Training, Strength[Title/Abstract]) OR (Aerobic Training[Title/Abstract]))	**Total**<br>23

	AND<br><br>(clinicalstudy[Filter] OR clinicaltrial[Filter] OR clinicaltrialphasei[Filter] OR clinicaltrialphaseii[Filter] OR clinicaltrialphaseiii[Filter] OR clinicaltrialphaseiv[Filter] OR controlledclinicaltrial[Filter] OR randomizedcontrolledtrial[Filter])	
**Embase**	('hypertrophic cardiomyopathy'/exp OR 'asymmetric septal hypertrophy' OR 'asymmetrical septal hypertrophy' OR 'cardiomyopathy, hypertrophic' OR 'hypertrophic cmp' OR 'hypertrophic cardiomyopathies' OR 'hypertrophic cardiomyopathy' OR 'hypertrophic myocardiopathies' OR 'hypertrophic myocardiopathy' OR 'hypertrophic obstructive cardiomyopathy'/exp OR 'cardiomyopathy, hypertrophic obstructive' OR 'cardiomyopathy, obstructive' OR 'hypertrophic cardiomyopathy, obstructive' OR 'hypertrophic obstructive cardiomyopathies' OR 'hypertrophic obstructive cardiomyopathy' OR 'hypertrophic obstructive myocardiopathy' OR 'myocardiopathy, obstructive' OR 'obstructive hcm' OR 'obstructive hcmp' OR 'obstructive cardiomyopathies' OR 'obstructive cardiomyopathy' OR 'obstructive hypertrophic cardiomyopathies' OR 'obstructive hypertrophic myocardiopathy' OR 'obstructive myocardiopathy' OR 'hypertrophic nonobstructive cardiomyopathy'/exp OR 'hypertrophic non-obstructive cardiomyopathy' OR 'hypertrophic nonobstructive cardiomyopathy' OR 'myocardiopathy, nonobstructive hypertrophic' OR 'non obstructive hypertrophic cardiomyopathy' OR 'non obstructive hypertrophic myocardiopathy' OR 'non-obstructive hcm' OR 'nonobstructive hcm' OR 'nonobstructive hypertrophic cardiomyopathy' OR 'nonobstructive hypertrophic myocardiopathy')<br><br>AND<br><br>('exercise'/exp OR 'biometric exercise' OR 'effort' OR 'exercise' OR 'exercise capacity' OR 'exercise performance' OR 'exercise training' OR 'exertion' OR 'fitness training' OR 'fitness workout' OR 'physical conditioning, human' OR 'physical effort' OR 'physical exercise' OR 'physical exertion' OR 'physical work-out' OR 'physical workout' OR 'resistance training'/exp OR 'resistance exercise' OR 'resistance exercise training' OR 'resistance training' OR 'resistance-type exercise' OR 'resistance-type training' OR 'strength training' OR 'strength-type exercise' OR 'strength-type training' OR 'aerobic training'/exp OR 'sport'/exp OR	**Total**<br>28

	'competitive gymnastics' OR 'competitive sport' OR 'sport' OR 'sports' OR 'athlete'/exp OR 'athlete' OR 'athletes' OR 'sportman' OR 'sportmen' OR 'sports player' OR 'sports players' OR 'sportsman' OR 'sportsmen' OR 'sportspeople' OR 'sportsperson' OR 'sportspersons' OR 'sportsplayers' OR 'sportswoman' OR 'sportswomen' OR 'sportwomen' OR 'training'/exp OR 'army training' OR 'athletic training' OR 'detraining' OR 'military training' OR 'physical training' OR 'sport specific training' OR 'technical training' OR 'training' OR 'training athlete' OR 'training, athletic' OR 'training, military' OR 'training, physical')<br><br>AND<br><br>('clinical study'/de OR 'clinical trial'/de OR 'clinical trial topic'/de OR 'controlled clinical trial'/de OR 'randomized controlled trial'/de OR 'randomized controlled trial topic'/de) AND 'article'/it<br><br>AND<br><br>[embase]/lim NOT ([embase]/lim AND [medline]/lim)	
**Cochrane Library**	#1 MeSH descriptor: [Cardiomyopathy, Hypertrophic] explode all trees<br><br>#2 hypertrophic cardiomyopathy or cardiomyopathy, hypertrophic obstructive or cardiomyopathy, hypertrophic non-obstructive or apical hypertrophic cardiomyopathy or septal hypertrophic cardiomyopathy or asymmetric septal hypertrophy or hypertrophic obstructive cardiomyopathy or hypertrophic non-obstructive cardiomyopathy or hypertrophic cardiomyopathies or cardiomyopathies, hypertrophic or cardiomyopathies, hypertrophic or obstructive cardiomyopathies<br><br>#3 #1 or #2<br><br>#4 MeSH descriptor: [Exercise] explode all trees<br><br>#5 MeSH descriptor: [Resistance Training] explode all trees	**Total**<br>168

#6 MeSH descriptor: [Physical Fitness] explode all trees

#7 MeSH descriptor: [Exercise Therapy] explode all trees

#8 MeSH descriptor: [High-intensity interval training] explode all trees

#9 exercises or resistance exercise or strength training or aerobic exercise or aerobic exercises or exercise aerobic or aerobic training or aerobic versus strength or strength versus aerobic or physical training or exercise therapy or physical exercise or combined training or aerobic plus strength training or aerobic plus resistance exercise or aerobic plus resistance training or concurrent training or concurrent exercise or HIIT or sprint interval training or vigorous exercise or sport or sports or isometric exercise or exercise, isometric or physical activities or athletic or team sports or team sport

#10 #4 or #5 or #6 or #7 or #8 or #9

#11 #3 and #10

In Trials

Tabela 2S. Estratégia de busca por estudos observacionais.

**PubMed/MEDLINE**	((Cardiomyopathy, Hypertrophic[MeSH Terms]) OR (Hypertrophic Cardiomyopathy[Title/Abstract]) OR (Hypertrophic Cardiomyopathies[Title/Abstract]) OR (Cardiomyopathies, Hypertrophic[Title/Abstract]) OR (Cardiomyopathy, Hypertrophic Obstructive[Title/Abstract]) OR (Cardiomyopathies, Hypertrophic Obstructive[Title/Abstract]) OR (Hypertrophic Obstructive Cardiomyopathies[Title/Abstract]) OR (Hypertrophic Obstructive Cardiomyopathy[Title/Abstract]) OR (Obstructive Cardiomyopathies, Hypertrophic[Title/Abstract]) OR (Obstructive Cardiomyopathy, Hypertrophic[Title/Abstract]) OR (Non-obstructive Hypertrophic Cardiomyopathy[Title/Abstract]) OR (Hypertrophic Non-obstructive Cardiomyopathy[Title/Abstract]) OR (Cardiomyopathy, Hypertrophic Non-obstructive[Title/Abstract]))<br><br>AND<br><br>((Exercise[MeSH Terms]) OR (Exercises[Title/Abstract]) OR (Exercise, Physical[Title/Abstract]) OR (Exercises, Physical[Title/Abstract]) OR (Physical Exercise[Title/Abstract]) OR (Physical Exercises[Title/Abstract]) OR (Physical Activity[Title/Abstract]) OR (Activities, Physical[Title/Abstract]) OR (Activity, Physical[Title/Abstract]) OR (Physical Activities[Title/Abstract]) OR (Exercise, Aerobic[Title/Abstract]) OR (Aerobic Exercise[Title/Abstract]) OR (Aerobic Exercises[Title/Abstract]) OR (Exercises, Aerobic[Title/Abstract]) OR (Exercise, Isometric[Title/Abstract]) OR (Exercises, Isometric[Title/Abstract]) OR (Isometric Exercises[Title/Abstract]) OR (Isometric Exercise[Title/Abstract]) OR (Acute Exercise[Title/Abstract]) OR (Acute Exercises[Title/Abstract]) OR (Exercise, Acute[Title/Abstract]) OR (Exercises, Acute[Title/Abstract]) OR (Exercise Training[Title/Abstract]) OR (Exercise Trainings[Title/Abstract]) OR (Training, Exercise[Title/Abstract]) OR (Trainings, Exercise[Title/Abstract]) OR (Sports[MeSH Terms]) OR (Sport[Title/Abstract]) OR (Athletics[Title/Abstract]) OR (Athletic[Title/Abstract]) OR (Team Sports[MeSHTerms]) OR (Team Sport[Title/Abstract]) OR (Resistance Training[MeSH Terms]) OR (Training, Resistance[Title/Abstract]) OR (Strength Training[Title/Abstract]) OR (Training, Strength[Title/Abstract]) OR (Aerobic Training[Title/Abstract]))<br><br>AND	**Total**<br>29

	(multicenterstudy[Filter] OR observationalstudy[Filter])	
**Embase**	(‘hypertrophic cardiomyopathy’/exp OR ‘asymmetric septal hypertrophy’ OR ‘asymmetrical septal hypertrophy’ OR ‘cardiomyopathy, hypertrophic’ OR ‘hypertrophic cmp’ OR ‘hypertrophic cardiomyopathies’ OR ‘hypertrophic cardiomyopathy’ OR ‘hypertrophic myocardiopathies’ OR ‘hypertrophic myocardiopathy’ OR ‘hypertrophic obstructive cardiomyopathy’/exp OR ‘cardiomyopathy, hypertrophic obstructive’ OR ‘cardiomyopathy, obstructive’ OR ‘hypertrophic cardiomyopathy, obstructive’ OR ‘hypertrophic obstructive cardiomyopathies’ OR ‘hypertrophic obstructive cardiomyopathy’ OR ‘hypertrophic obstructive myocardiopathy’ OR ‘myocardiopathy, obstructive’ OR ‘obstructive hcm’ OR ‘obstructive hcmp’ OR ‘obstructive cardiomyopathies’ OR ‘obstructive cardiomyopathy’ OR ‘obstructive hypertrophic cardiomyopathies’ OR ‘obstructive hypertrophic myocardiopathy’ OR ‘obstructive myocardiopathy’ OR ‘hypertrophic nonobstructive cardiomyopathy’/exp OR ‘hypertrophic non-obstructive cardiomyopathy’ OR ‘hypertrophic nonobstructive cardiomyopathy’ OR ‘myocardiopathy, nonobstructive hypertrophic’ OR ‘non obstructive hypertrophic cardiomyopathy’ OR ‘non obstructive hypertrophic myocardiopathy’ OR ‘non-obstructive hcm’ OR ‘nonobstructive hcm’ OR ‘nonobstructive hypertrophic cardiomyopathy’ OR ‘nonobstructive hypertrophic myocardiopathy’)<br><br>AND<br><br>(‘exercise’/exp OR ‘biometric exercise’ OR ‘effort’ OR ‘exercise’ OR ‘exercise capacity’ OR ‘exercise performance’ OR ‘exercise training’ OR ‘exertion’ OR ‘fitness training’ OR ‘fitness workout’ OR ‘physical conditioning, human’ OR ‘physical effort’ OR ‘physical exercise’ OR ‘physical exertion’ OR ‘physical work-out’ OR ‘physical workout’ OR ‘resistance training’/exp OR ‘resistance exercise’ OR ‘resistance exercise training’ OR ‘resistance training’ OR ‘resistance-type exercise’ OR ‘resistance-type training’ OR ‘strength training’ OR ‘strength-type exercise’ OR ‘strength-type training’ OR ‘aerobic training’/exp OR ‘sport’/exp OR ‘competitive gymnastics’ OR ‘competitive sport’ OR ‘sport’ OR ‘sports’ OR ‘athlete’/exp OR ‘athlete’ OR ‘athletes’ OR ‘sportman’ OR ‘sportmen’ OR ‘sports player’ OR ‘sports players’ OR ‘sportsman’ OR ‘sportsmen’ OR ‘sportspeople’ OR ‘sportsperson’ OR ‘sportspersons’ OR ‘sportsplayers’ OR ‘sportswoman’ OR ‘sportswomen’ OR ‘sportwomen’ OR ‘training’/exp OR ‘army training’ OR ‘athletic training’ OR	**Total**<br>132

	‘detraining’ OR ‘military training’ OR ‘physical training’ OR ‘sport specific training’ OR ‘technical training’ OR ‘training’ OR ‘training athlete’ OR ‘training, athletic’ OR ‘training, military’ OR ‘training, physical’)<br><br>AND<br><br>(‘cohort analysis’/de OR ‘control group’/de OR ‘longitudinal study’/de OR ‘multicenter study’/de OR ‘observational study’/de OR ‘prospective study’/de OR ‘retrospective study’/de) AND ‘article’/it<br><br>AND<br><br>[embase]/lim NOT ([embase]/lim AND [medline]/lim)	
**Cochrane Library**	#1 MeSH descriptor: [Cardiomyopathy, Hypertrophic] explode all trees<br><br>#2 hypertrophic cardiomyopathy or cardiomyopathy, hypertrophic obstructive or cardiomyopathy, hypertrophic non-obstructive or apical hypertrophic cardiomyopathy or septal hypertrophic cardiomyopathy or asymmetric septal hypertrophy or hypertrophic obstructive cardiomyopathy or hypertrophic non-obstructive cardiomyopathy or hypertrophic cardiomyopathies or cardiomyopathies, hypertrophic or cardiomyopathies, hypertrophic or obstructive cardiomyopathies<br><br>#3 #1 or #2<br><br>#4 MeSH descriptor: [Exercise] explode all trees<br><br>#5 MeSH descriptor: [Resistance Training] explode all trees<br><br>#6 MeSH descriptor: [Physical Fitness] explode all trees<br><br>#7 MeSH descriptor: [Exercise Therapy] explode all trees	**Total**<br>75

#8 MeSH descriptor: [High-intensity interval training] explode all trees

#9 exercises or resistance exercise or strength training or aerobic exercise or aerobic exercises or exercise aerobic or aerobic training or aerobic versus strength or strength versus aerobic or physical training or exercise therapy or physical exercise or combined training or aerobic plus strength training or aerobic plus resistance exercise or aerobic plus resistance training or concurrent training or concurrent exercise or HIIT or sprint interval training or vigorous exercise or sport or sports or isometric exercise or exercise, isometric or physical activities or athletic or team sports or team sport

#10 #4 or #5 or #6 or #7 or #8 or #9

#11 #3 and #10

#12 cohort analysis OR control group OR longitudinal study OR multicenter study OR observational study OR prospective study OR retrospective study

#13 #3 and #10 and #12

Tabela 3S. Lista de estudos potencialmente elegíveis excluídos.

Primeiro autor	Título	Revista	Ano	Razão para exclusão
Aengevaeren, VL	Association between Lifelong Physical Activity and Disease Characteristics in HCM	Med Sci Sports Exerc	2019	Ausência de grupo comparador de interesse
Basu, J	Safety and outcomes of a high intensity exercise programme in young patients with hypertrophic cardiomyopathy: The SAFE-HCM trial	Heart	2021	Pôster de congresso
Basu, J	Six-month outcomes of a high intensity exercise programme in young patients with hypertrophic cardiomyopathy: The SAFE-HCM trial	Eur J Prev Cardiol	2021	Pôster de congresso
Sadowski, K	Hybrid telerehabilitation in patients with hypertrophic cardiomyopathy without left ventricular outflow tract obstruction and preserved left ventricular ejection fraction-the randomised clinical trial	Eur Heart J Digit Health	2021	Pôster de congresso

Tabela 4S. Características demográficas dos pacientes dos estudos incluídos.

Estudo	Variante patogênica ou provavelmente patogênica	Realce Tardio	FEVE (%)	Trato da via de saída do VE (média)	Histórico prévio de exercício
Basu et al.[13]	28% (grupo exercício)<br><br>28% (grupo controle)	NR	65	Repouso:<br>4,5 mmHg (grupo exercício)<br><br>5,0 mmHg (grupo controle)<br><br>Após provocação:<br>6,8 mmHg (grupo exercício)<br><br>12,0 mmHg (grupo controle)	Coletaram história de exercício, mas não há essa informação disponível.
Gudmundsdottiret al.[20]	34% (grupo exercício)<br><br>50% (grupo controle)	NR	58,5	Repouso:<br>9 mmHg (grupo exercício)<br><br>8 mmHg (grupo controle)<br><br>Após exercício:<br>19 mmHg (grupo exercício)<br><br>18 mmHg (grupo controle)	Participantes que realizavam >1 h/semana de exercício moderado-vigoroso foram excluídos. Nível detalhado de exercício prévio não foi descrito.
MaCnamara et al.[12]	NR	NR	64,5	Repouso:<br>10 mmHg (grupo moderado)<br><br>14 mmHg (grupo intenso)<br><br>Após Valsalva:<br>14 mmHg (grupo moderado)	Atletas e indivíduos que já realizavam treinamento de alta intensidade foram excluídos. Nível detalhado de exercício prévio não foi descrito.

				23 mmHg (grupo intenso)	
Saberi et al.[11]	49,3% (grupo exercício)<br><br>36,2% (grupo controle)	% da massa do VE:<br>3,1% (grupo exercício)<br><br>2,9% (grupo controle)	70,7	Repouso:<br>15,2 mmHg (grupo exercício)<br><br>20,7 mmHg (grupo controle)<br><br>Após Valsalva:<br>23,6 mmHg (grupo exercício)<br><br>31,8 mmHg (grupo controle)<br><br>Após exercício:<br>48,1 mmHg (grupo exercício)<br><br>57,0 mmHg (grupo controle)	Aplicaram questionário (frequência semanal e duração das sessões no mês anterior). Os resultados não foram descritos.
Kwon et al.[22]	NR	NR	NR	NR	Questionário de atividade física (últimos 7 dias); frequência de atividades leves, moderadas e vigorosas. Nenhuma exclusão baseada no nível prévio de exercício.
Lampert et al.[21]	44,4% (sedentários)<br><br>42,7% (moderadamente ativos)<br><br>47,2% (vigorosamente ativos)	Leve/moderado (<15%):<br>16,2% (sedentários)<br><br>18,3% (moderadamente ativos)<br><br>17,6% (vigorosamente ativos)	66	Repouso:<br>24,7 mmHg (sedentários; ≥35 mmHg: 21,8%)<br><br>22,4 mmHg (moderadamente ativos; ≥35 mmHg: 18,8%)<br><br>18,3 mmHg (vigorosamente ativos;	Nível de atividade física prévia: 15% sedentários, 43% moderados e 42% vigorosos, avaliado pelo Minnesota Leisure Time Activity Questionnaire (atividade física no último ano).

		Extenso (>15%):<br>6,5% (sedentários)<br><br>6,2% (moderadamente ativos)<br><br>6,9% (vigorosamente ativos)		≥35 mmHg: 11,9%)<br><br>Após Valsalva ou exercício:<br>50,2 mmHg (sedentários;<br>≥35 mmHg: 51,4%)<br><br>51,4 mmHg (moderadamente ativos;<br>≥35 mmHg: 51,5%)<br><br>39,6 mmHg (vigorosamente ativos;<br>≥35 mmHg: 37,8%)	
Martinez et al.[24]	NR	NR	NR	NR	Histórico competitivo de alto rendimento (atletas NCAA Divisão I, olímpicos ou profissionais). Volume prévio de treinamento não descrito.
Pelliccia et al.[23]	13/27 (48%)	22% (atletas treinados)<br><br>39% (atletas destreinados)	65	NR	Atletas competitivos; mediana de 7 h/semana de treinamento e 14 anos de prática competitiva.

CMH: cardiomiopatia hipertrófica; FEVE: fração de ejeção do ventrículo esquerdo; NCAA: National Collegiate Athletic Association; NR: não reportado; NYHA: New York Heart Association; VE: ventrículo esquerdo.

Percentual de atletas com realce tardio no estudo de Pelliccia et al.[23]: baseado em dados disponíveis de 71 dos 88 atletas.

Tabela 5S. Critérios de inclusão e exclusão dos estudos incluídos.

Estudo	Critérios de Inclusão	Critérios-chave para exclusão
Basu et al.[13]	1) Diagnóstico de CMH (espessura máxima da parede do VE ≥15 mm, na ausência de condições de sobrecarga anormal); 2) Idade entre 16–60 anos; 3) Classe funcional I ou II da NYHA; 4) Capacidade de realizar EF; 5) Capacidade de se comprometer com a duração do programa de treinamento definido pelo protocolo.	1) Atletas competitivos; 2) Histórico de síncope induzida por exercício; 3) Arritmias ventriculares mal controladas; 4) Miectomia cirúrgica; 5) FEVE severamente reduzida (<35%); 6) Gradiente no trato de saída do VE ≥50 mmHg em repouso, após Valsalva ou após agachamento; 7) Aguardando ou com implante recente de dispositivo (< 3 meses); 8) Doença arterial coronariana conhecida (lesão >50% na angiografia coronariana ou intervenção coronariana prévia); 9) Insuficiência renal; 10) Fenocópias de CMH; 11) Gravidez.
Gudmundsdottir et al.[20]	1) Adultos (≥18 anos); 2) Diagnóstico fenotípico confirmado de CMH (espessura máxima da parede do VE ≥15 mm, na ausência de outras causas primárias de hipertrofia do VE; ou espessura máxima da parede do VE ≥13 mm em indivíduos com parente de primeiro grau portador de uma variante genética definitivamente ou provavelmente causadora da doença).	1) Gradiente no trato de saída do VE ≥30 mmHg em repouso ou ≥ 50 mmHg após Valsalva; 2) História de síncope induzida por exercício, arritmias ventriculares ou angina grave (classe funcional III-IV); 3) Disfunção valvar com repercussão hemodinâmica significativa; 4) Menos de 3 meses após terapia de redução septal; 5) Alterações recentes na medicação que influenciem a resposta ao exercício ou a hemodinâmica (ex.: betabloqueadores, bloqueadores dos canais de cálcio); 6) Gravidez.
MaCnamara et al.[12]	1) Idade entre 18–65 anos; 2) Diagnóstico de CMH (hipertrofia do VE com espessura diastólica final da parede ≥15 mm, ou espessura entre 13–15 mm associada a história familiar de CMH ou teste genético positivo). A hipertrofia deveria ocorrer na ausência de doença sistêmica capaz de explicar o achado ou ser desproporcional ao grau de hipertrofia esperado pela doença sistêmica; 3) Pacientes com CDI previamente implantado puderam ser incluídos.	1) Arritmias induzidas pelo exercício; 2) Obstrução do trato de saída do VE ≥ 50 mmHg em repouso apesar da terapia medicamentosa; 3) Terapia de redução septal recente; 4) Gravidez; 5) Sintomas (classe IV da NYHA); 6) Resposta hipotensiva ao exercício (queda da pressão arterial sistólica > 20 mmHg em relação ao valor de repouso ou durante o exercício); 7) Disfunção sistólica do VE; 8) Infarto do miocárdio ou acidente vascular cerebral prévios, ou incapacidade para realizar EF.
Saberi et al.[11]	1) Idade entre 18–80 anos; 2) Diagnóstico de CMH (espessura diastólica final da parede do VE ≥15 mm ao ecocardiograma bidimensional, na ausência de outras causas primárias de hipertrofia do VE; ou espessura entre 13–15 mm na presença de características sugestivas de CMH, como:	1) Histórico de síncope induzida por exercício ou arritmias ventriculares; 2) Obstrução do trato de saída do VE refratária ao tratamento clínico em avaliação para terapia de redução septal; 3) Menos de 3 meses após realização de terapia de redução septal ou implante de CDI; 4) Histórico de resposta

	movimento sistólico anterior da valva mitral; história familiar de CMH; teste genético positivo).	hipotensiva durante teste de esforço (definida como queda da pressão arterial sistólica >20 mmHg em relação ao valor basal, ou aumento inicial seguido de queda >20 mmHg); 5) Descompensação clínica nos 3 meses anteriores, definida como sintomas de insuficiência cardíaca congestiva (classe IV da NYHA) ou angina classe IV da Canadian Cardiovascular Society; 6) FEVE menor que 55% na ecocardiografia; 7) Expectativa de vida inferior a 12 meses; 8) Gravidez.
Kwon et al.[22]	1) Indivíduos com diagnóstico de CMH; 2) Realização de avaliação de saúde dentro de 1 ano após o diagnóstico de CMH; 3) Disponibilidade completa dos dados das variáveis analisadas; 4) Relato de algum nível de atividade física no questionário da avaliação de saúde.	Indivíduos que relataram nenhuma prática de EF nos questionários de avaliação de saúde, devido à preocupação de que esses indivíduos apresentassem comorbidades graves que os impedissem de realizar EF.
Lampert et al.[21]	1) Idade entre 8–60 anos; 2) Presença de CMH manifesta (fenótipo positivo) ou portadores de variante genética associada à CMH sem expressão fenotípica (genótipo positivo/fenótipo negativo).	1) Condições que contraindiquem exercício vigoroso (por exemplo, sintomas avançados relacionados à CMH, como classe III ou IV da NYHA, ou condições não relacionadas à CMH); 2) Hipertrofia do VE secundária a condições sindrômicas ou doenças infiltrativas; 3) Indivíduos incapazes de responder questionários *on-line* ou por telefone devido a barreiras linguísticas ou cognitivas.
Martinez et al.[24]	1) Atletas de elite (*National Collegiate Athletic Association*, primeira divisão, olímpicos ou profissionais); 2) Com doença cardíaca genética associada ao risco de morte súbita, avaliados e liberados formalmente para retorno ao esporte em um dos quatro centros especializados participantes.	NR.
Pelliccia et al.[23]	1) Atletas com CMH que participavam regularmente de programas de EF e esporte competitivo; 2) CMH definida por espessura da parede do VE ≥15 mm, ou 13–14 mm associada a história familiar positiva, alterações eletrocardiográficas significativas e/ou mutação genética patogênica.	NR.

CDI: Cardiodesfibrilador implantável; CMH: Cardiomiopatia hipertrófica; EF: exercício físico; FEVE: Fração de ejeção do ventrículo esquerdo; NR: não reportado; NYHA: New York Heart Association; VE: Ventrículo esquerdo.

Tabela 6S. Características das intervenções dos estudos incluídos.

Estudo	Desenho	Duração/ seguimento	Intervenção	Volume	Intensidade	Grupo comparador
Basu et al.[13]	ECR	12 semanas	Treinamento de alta intensidade	Sessões supervisionadas de 1 hora, 2x/semana, além de 1h/semana de exercícios domiciliares. O programa incluía aquecimento, circuito alternado de exercícios aeróbicos e de resistência (pelo menos 50% do tempo dedicado a exercícios aeróbicos) e desaquecimento. Um programa com níveis graduais permitia progressão individualizada. Sessões adicionais em casa eram realizadas com suporte de programa pré-gravado e materiais impressos	Iniciado a 70% da FC de reserva, progredindo em incrementos de 5% até 85% da FC de reserva. Para pacientes com CDI, o alvo era pelo menos 10 bpm abaixo do limiar de ativação do dispositivo. A percepção de esforço foi monitorada pela escala de Borg, e houve monitoramento contínuo por ECG durante as sessões supervisionadas	Atividades usuais
Gudmundsdottir et al.[20]	ECR	12 semanas	Treinamento de intensidade moderada	Sessões de 60 min, 3x/semana. Cada sessão incluiu 10 min de aquecimento, 30 min de treino intervalado com bicicleta ergométrica e 20 min de fortalecimento muscular, supervisionados por fisioterapeuta especializado	60% da capacidade máxima de trabalho; escala de Borg 12-14	Atividades usuais
MaCnamara et al.[12]	ECR	5 meses	Treinamento de intensidade moderada e de alta intensidade	Grupo moderado: no primeiro mês, sessões de 30 min na zona de *base pace* (ritmo leve a moderado, FC 1-20 bpm abaixo do *maximal steady state*, que	Grupo moderado: FC entre 1 e 20 bpm abaixo do "maximal steady state"	-

				corresponde a uma intensidade próxima ao limiar ventilatório). Após isso, aumento gradual até 1 sessão de 60 min e 3 sessões de 30 min/semana, além de 1 sessão de recuperação semanal<br><br>Grupo intenso: no primeiro mês, sessões iguais ao grupo moderado (30 min na zona de *base pace*). A partir do segundo mês, iniciaram sessões em *maximal steady state* e, no terceiro mês, passaram a realizar 14-15 sessões intervaladas de 4×4 min, com 3 min de descanso ativo (~70% da FC máxima). Todas as sessões foram supervisionadas com monitoramento contínuo por ECG	Grupo intenso: sessões intervaladas que incluíam 10 min de aquecimento, seguidas por intervalos a 90-95% da FC máxima, intercalados por recuperação ativa a cerca de 70% da FC máxima, além de sessões em *maximal steady state* (limiar ventilatório ± 5 bpm)	
Saberi et al.[11]	ECR	16 semanas	Treinamento de intensidade moderada	Programa estruturado (não supervisionado) com pelo menos 3 sessões/semana, iniciando com 20 min por sessão. A duração foi aumentando semanalmente entre 5-10 min, até 60 min/sessão, com uma frequência de 4-7 sessões/semana. Os modos de exercício incluíram ciclismo, caminhada/corrida leve e treinamento elíptico. Não foram prescritos exercícios de fortalecimento muscular ou atividades explosivas. Os participantes receberam uma consulta de 1 h com um fisiologista do exercício certificado	11-14 na escala de Borg. Durante o primeiro mês, a intensidade foi progressivamente aumentada para atingir 70% da FC de reserva, mantendo essa intensidade nas semanas 5 a 16 do protocolo	Atividades usuais

Kwon et al.[22]	Observacional	5,3 anos	Grupo 1 (tercil mais baixo): participantes com o menor nível de AF<br><br>Grupo 2 (tercil médio): participantes com nível moderado de AF<br><br>Grupo 3 (tercil mais alto): participantes com o maior nível de AF	Grupo 1: participantes com o menor volume de AF diária, correspondendo ao tercil mais baixo do *Physical Activity Score*† em METs/dia, indicando baixa frequência e/ou intensidade das atividades relatadas<br><br>Grupo 2: participantes com volume moderado de AF diária, correspondendo ao tercil médio do *Physical Activity Score*†, caracterizando prática regular de atividades leves e moderadas<br><br>Grupo 3: participantes com o maior volume de EF diário, correspondendo ao tercil mais alto do *Physical Activity Score*†, refletindo prática mais frequente e/ou intensa, incluindo atividades moderadas a vigorosas	Grupo 1: engajado principalmente em atividades de intensidade leve (≈3 METs)<br><br>Grupo 2: engajado em uma combinação de atividades leves e moderadas (≈3-5 METs)<br><br>Grupo 3: engajado regularmente em atividades de intensidade moderada a vigorosa (≈5-8 METs)	AF de baixa intensidade
Lampert et al.[21]	Observacional	3,2 anos	Atividade de intensidade moderada ou vigorosa	Grupo vigoroso: participação em pelo menos uma atividade com intensidade de 6,0 METs ou mais, totalizando 60 h ou mais por ano<br><br>Grupo moderado: participação em atividades com intensidade entre 4,0 e 5,9 METs, por 60 h ou mais por ano, sem atender aos critérios para exercício vigoroso	Grupo vigoroso: atividades com intensidade igual ou superior a 6,0 METs<br><br>Grupo moderado: atividades com intensidade entre 4,0 e 5,9 METs	Grupo sedentário: atividades com intensidade inferior a 4,0 METs

Martinez et al.[24]	Observacional	7 anos	Retorno à prática esportiva de elite	NR (presumivelmente alto, dado o nível competitivo)	NR (presumivelmente alta, dado o nível competitivo)	-
Pelliccia et al.[23]	Observacional	7 anos	Atletas com manutenção do treinamento de alta intensidade	Atletas com manutenção do treinamento: 3 (ou mais) sessões/semana, com duração de 2 h ou mais por sessão, incluindo exercício de alta intensidade, totalizando ≥6 até 14 h de treinamento/semana	Sessões de exercício de alta intensidade	Atletas que interromperam os treinamentos (sedentários) ou com prática ocasional de lazer (<6 h/semana)

ECR: ensaio clínico randomizado; EF: exercício físico; FC: frequência cardíaca; NR: não reportado; MET = equivalente metabólico.

† Representa a média diária de gasto energético em METs, calculada a partir da frequência semanal de AFs leves, moderadas e vigorosas.

Tabela 7S. Conflitos de interesse, DOIs, registro e suporte financeiro dos estudos.

Estudo	Revista	DOI	Registro	Financiamento	Conflitos de interesse
Basu et al.[13]	EHJ	10.1093/eurheartj/ehae919	NCT05459467	Sim	Sim
Gudmundsdottir et al.[20]	Circulation	10.1161/CIRCULATIONAHA.124.070064	NCT03537183	Sim	Não
MacNamara et al.[12]	JAHA	10.1161/JAHA.123.031399	NCT03335332	Sim	Não
Saberi et al.[11]	JAMA	10.1001/jama.2017.2503	NCT01127061	Sim	Não
Kwon et al.[22]	BJSM	10.1136/bjsports-2020-101987	NR	Não	Sim
Lampert et al.[21]	JAMA Cardiology	10.1001/jamacardio.2023.1042	NR	Sim	Sim
Martinez et al.[24]	JACC	10.1016/j.jacc.2023.05.059	NR	Sim	Sim
Pelliccia et al.[23]	BJSM	10.1136/bjsports-2019-100890	NR	Não	Não

BJSM: British Journal of Sports Medicine; EHJ: European Heart Journal; JACC: Journal of the American College of Cardiology; JAHA: Journal of the American Heart Association; JAMA: Journal of the American Medical Association; NR: não reportado.

Tabela 8S. Checklist PRISMA da revisão sistemática.

Seção e Tópico	Item nº	Item da Lista de Verificação	Local onde o item está relatado
**TÍTULO**			
Título	1	Identificar o relatório como uma revisão sistemática.	8
**RESUMO**			
Resumo	2	Veja a checklist PRISMA 2020 para resumos (PRISMA 2020 for Abstracts).	6
**INTRODUÇÃO**			
Racional	3	Descrever a justificativa para a revisão no contexto do conhecimento existente.	7
Objetivos	4	Fornecer uma declaração explícita do(s) objetivo(s) ou da(s) pergunta(s) que a revisão aborda.	7
**MÉTODOS**			
Critérios de elegibilidade	5	Especificar os critérios de inclusão e exclusão da revisão e como os estudos foram agrupados para as sínteses.	9
Fontes de informação	6	Especificar todas as bases de dados, registros, sites, organizações, listas de referências e outras fontes pesquisadas ou consultadas para identificar os estudos. Informar a data em que cada fonte foi pesquisada ou consultada pela última vez.	9
Estratégia de busca	7	Apresentar as estratégias completas de busca para todas as bases de dados, registros e sites, incluindo quaisquer filtros e limites utilizados.	MAT SUP 1S e 2S
Processo de seleção	8	Especificar os métodos utilizados para decidir se um estudo atendia aos critérios de inclusão da revisão, incluindo quantos avaliadores fizeram a triagem de cada registro e de cada relatório obtido, se trabalharam de forma independente e, quando aplicável, detalhes sobre as ferramentas automatizadas usadas no processo.	10
Processo de coleta de dados	9	Especificar os métodos usados para coletar dados dos relatórios, incluindo quantos avaliadores coletaram dados de cada relatório, se trabalharam de forma independente, quaisquer processos para obtenção ou confirmação de dados junto aos investigadores dos estudos e, quando aplicável, detalhes sobre as ferramentas automatizadas utilizadas no processo.	10
Itens de dados	10a	Listar e definir todos os desfechos para os quais os dados foram buscados. Especificar se foram buscados todos os resultados compatíveis com cada domínio de desfecho em cada estudo (por exemplo, para todas as medidas, momentos de avaliação, análises) e, caso contrário, os métodos utilizados para decidir quais resultados coletar.	10
	10b	Listar e definir todas as outras variáveis para as quais os dados foram buscados (por exemplo, características dos participantes e das intervenções, fontes de financiamento). Descrever quaisquer suposições feitas sobre informações faltantes ou pouco claras.	10

Seção e Tópico	Item nº	Item da Lista de Verificação	Local onde o item está relatado
Avaliação do risco de viés dos estudos	11	Especificar os métodos utilizados para avaliar o risco de viés nos estudos incluídos, incluindo detalhes das ferramentas utilizadas, quantos avaliadores realizaram a avaliação de cada estudo e se trabalharam de forma independente, e, quando aplicável, detalhes sobre ferramentas automatizadas utilizadas no processo.	10
Medidas de efeito	12	Especificar, para cada desfecho, a(s) medida(s) de efeito (por exemplo, razão de risco, diferença média) utilizada(s) na síntese ou na apresentação dos resultados.	10
Métodos de síntese	13a	Descrever os processos utilizados para decidir quais estudos foram elegíveis para cada síntese (por exemplo, tabular as características das intervenções dos estudos e compará-las com os grupos planejados para cada síntese — item nº 5).	10
	13b	Descrever quaisquer métodos necessários para preparar os dados para apresentação ou síntese, como o tratamento de estatísticas sumárias ausentes ou conversões de dados.	10
	13c	Descrever quaisquer métodos utilizados para tabular ou exibir visualmente os resultados dos estudos individuais e das sínteses.	10
	13d	Descrever quaisquer métodos utilizados para sintetizar os resultados e apresentar a justificativa para a(s) escolha(s). Caso tenha sido realizada metanálise, descrever o(s) modelo(s) utilizado(s), os métodos para identificar a presença e a extensão da heterogeneidade estatística, e o(s) pacote(s) de software empregado(s).	10
	13e	Descrever quaisquer métodos utilizados para explorar possíveis causas da heterogeneidade entre os resultados dos estudos (por exemplo, análises de subgrupos, metarregressão).	10
	13f	Descrever quaisquer análises de sensibilidade realizadas para avaliar a robustez dos resultados sintetizados.	10
Avaliação do viés de relato	14	Descrever quaisquer métodos utilizados para avaliar o risco de viés devido a resultados ausentes em uma síntese (decorrentes de vieses de relato).	NR
Avaliação da certeza (ou qualidade) da evidência	15	Descrever quaisquer métodos utilizados para avaliar a certeza (ou confiança) no conjunto de evidências para um desfecho.	10
**RESULTADOS**			
Seleção dos estudos	16a	Descrever os resultados do processo de busca e seleção, desde o número de registros identificados na busca até o número de estudos incluídos na revisão, preferencialmente utilizando um diagrama de fluxo.	11
	16b	Citar os estudos que poderiam parecer atender aos critérios de inclusão, mas que foram excluídos, e explicar o motivo da exclusão.	11 e 12
Características dos estudos	17	Citar cada estudo incluído e apresentar suas características.	Tabela 1

Seção e Tópico	Item nº	Item da Lista de Verificação	Local onde o item está relatado
Risco de viés nos estudos	18	Apresentar as avaliações do risco de viés para cada estudo incluído.	Figuras 1 e 2
Resultados dos estudos individuais	19	Para todos os desfechos, apresentar, para cada estudo: (a) estatísticas resumidas para cada grupo (quando apropriado) e (b) uma estimativa do efeito e sua precisão (por exemplo, intervalo de confiança ou credibilidade), preferencialmente utilizando tabelas estruturadas ou gráficos.	13 e 14
Resultados das sínteses	20a	Para cada síntese, resumir brevemente as características e o risco de viés dos estudos que contribuíram para ela.	13 e 14
	20b	Apresentar os resultados de todas as sínteses estatísticas realizadas. Se foi feita metanálise, apresentar para cada uma a estimativa resumida e sua precisão (por exemplo, intervalo de confiança ou credibilidade) e as medidas de heterogeneidade estatística. Caso haja comparação entre grupos, descrever a direção do efeito.	NR
	20c	Apresentar os resultados de todas as investigações sobre possíveis causas da heterogeneidade entre os resultados dos estudos.	20
	20d	Apresentar os resultados de todas as análises de sensibilidade realizadas para avaliar a robustez dos resultados sintetizados.	NR
Vieses de relato	21	Apresentar as avaliações do risco de viés devido a resultados ausentes (decorrentes de vieses de relato) para cada síntese avaliada.	NR
Certeza da evidência	22	Apresentar as avaliações da certeza (ou confiança) no conjunto de evidências para cada desfecho avaliado.	2-4
**DISCUSSÃO**			
Discussão	23a	Fornecer uma interpretação geral dos resultados no contexto de outras evidências.	16 a 19
	23b	Discutir quaisquer limitações das evidências incluídas na revisão.	19
	23c	Discutir quaisquer limitações dos processos utilizados na revisão.	19 e 20
	23d	Discutir as implicações dos resultados para a prática clínica, políticas públicas e pesquisas futuras.	20
**OUTRAS INFORMAÇÕES**			
Registro e protocolo	24a	Fornecer as informações de registro da revisão, incluindo o nome do registro e o número de registro, ou informar que a revisão não foi registrada.	8
	24b	Indicar onde o protocolo da revisão pode ser acessado, ou informar que um protocolo não foi elaborado.	8
	24c	Descrever e explicar quaisquer alterações feitas nas informações fornecidas no registro ou no protocolo.	NR
Suporte	25	Descrever as fontes de apoio financeiro ou não financeiro para a revisão, assim como o papel dos financiadores ou patrocinadores na condução da revisão.	11

Seção e Tópico	Item nº	Item da Lista de Verificação	Local onde o item está relatado
Conflitos de interesse	26	Declarar quaisquer conflitos de interesse dos autores da revisão.	11
Disponibilidade de dados, códigos e outros materiais	27	Informar quais dos seguintes materiais estão disponíveis publicamente e onde podem ser encontrados: formulários de coleta de dados padronizados; dados extraídos dos estudos incluídos; dados utilizados em todas as análises; códigos analíticos; quaisquer outros materiais usados na revisão.	Tabelas MAT SUP

Legenda: NR = NÃO REPORTADO; MAT SUP = Material Siplementar.

Tabela 9S. Eventos relacionados a arritmias, choques de CDI e síncopes nos estudos incluídos.

Estudo	TVS (N)	TVNS (N)	Choque de CDI (N)	Síncope (N)	Outros (N)
Basu et al.[13]	GC: 1<br><br>Grupo EF: 0	NR[¥]	NR	GC: 0<br><br>Grupo EF: 1	-
Gudmundsdottir et al.[20]	0	NR	0	NR	Palpitações:<br>Grupo EF: 1 (ritmo sinusal no ECG; sem evento no CDI)
MacNamara et al.[12]	0	Grupo EF moderado:<br>2 (treino); 3 (pós-treino)<br><br>Grupo EF intenso:<br>2 (treino); 1 (pós-treino)	0	0	TSV:<br>Grupo EF moderado:<br>1 (treino); 1 (pós-treino)<br><br>Grupo EF intenso:<br>0 (treino); 1 (pós-treino)
Saberi et al.[11]	0	GC: 15<br><br>Grupo EF: 19	0	GC: 2<br><br>Grupo EF: 0	TSV:<br>Grupo controle: 29<br><br>Grupo EF: 23<br><br>FA:<br>Grupo controle: 7<br><br>Grupo EF: 5
Kwon et al.[22]	NR	NR	NR	NR	-

Lampert et al.[21]	NR	NR	Sedentários: 3<br><br>Moderadamente ativos: 3<br><br>Vigorosamente ativos: 8	Com CDI:<br>Sedentários: 6<br><br>Moderadamente ativos: 13<br><br>Vigorosamente ativos: 15<br><br>Sem CDI:<br>Sedentários: 3<br><br>Moderadamente ativos: 5<br><br>Vigorosamente ativos: 7	-
Martinez et al.[24]	NR	NR	1[⁋]	1[#]	-
Pelliccia et al.[23]	NR	Atletas treinados: 6<br><br>Atletas destreinados: 19	NR	Atletas treinados: 4<br><br>Atletas destreinado: 5	FA paroxística:<br>Atletas destreinados: 2<br><br>Atletas treinados: 0

CDI: cardioversor-desfibrilador implantável; EF: exercício físico; FA: fibrilação atrial; GC: grupo controle; NR: não reportado; TVNS: taquicardia ventricular não sustentada; TSV: taquicardia supraventricular; TVS: taquicardia ventricular sustentada.

[⁋] Episódio não relacionado ao EF (ao mover móveis).

[#] O mesmo atleta teve dois episódios de síncope: um presumivelmente não arrítmico e outro durante o esforço.

[¥] Dez participantes receberam implante de CDI, dos quais sete tiveram como principal motivo episódios novos ou repetidos de TVNS. No entanto, a distribuição desses casos entre os grupos exercício e controle não foi detalhada.

Figura 1S. Fluxograma PRISMA da seleção de ensaios clínicos randomizados.

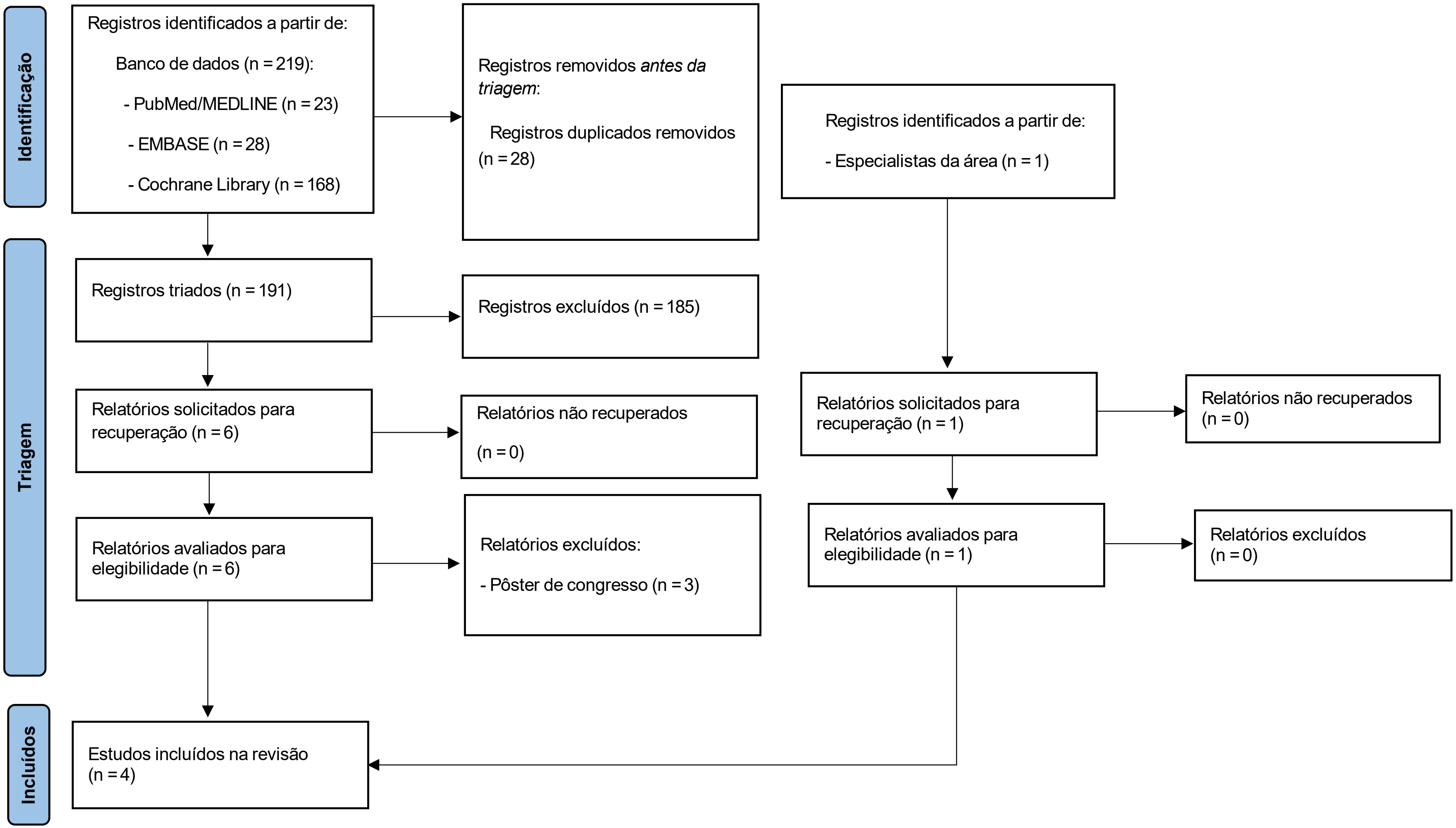

Obs.: o artigo de Basu et al.[13] (ECR) foi inicialmente recuperado na forma de resumo de congresso de 2021 e, posteriormente, incluído em sua versão completa, publicada em 2025, com o auxílio dos especialistas consultados.

Figura 2S. Fluxograma PRISMA da seleção de estudos observacionais.

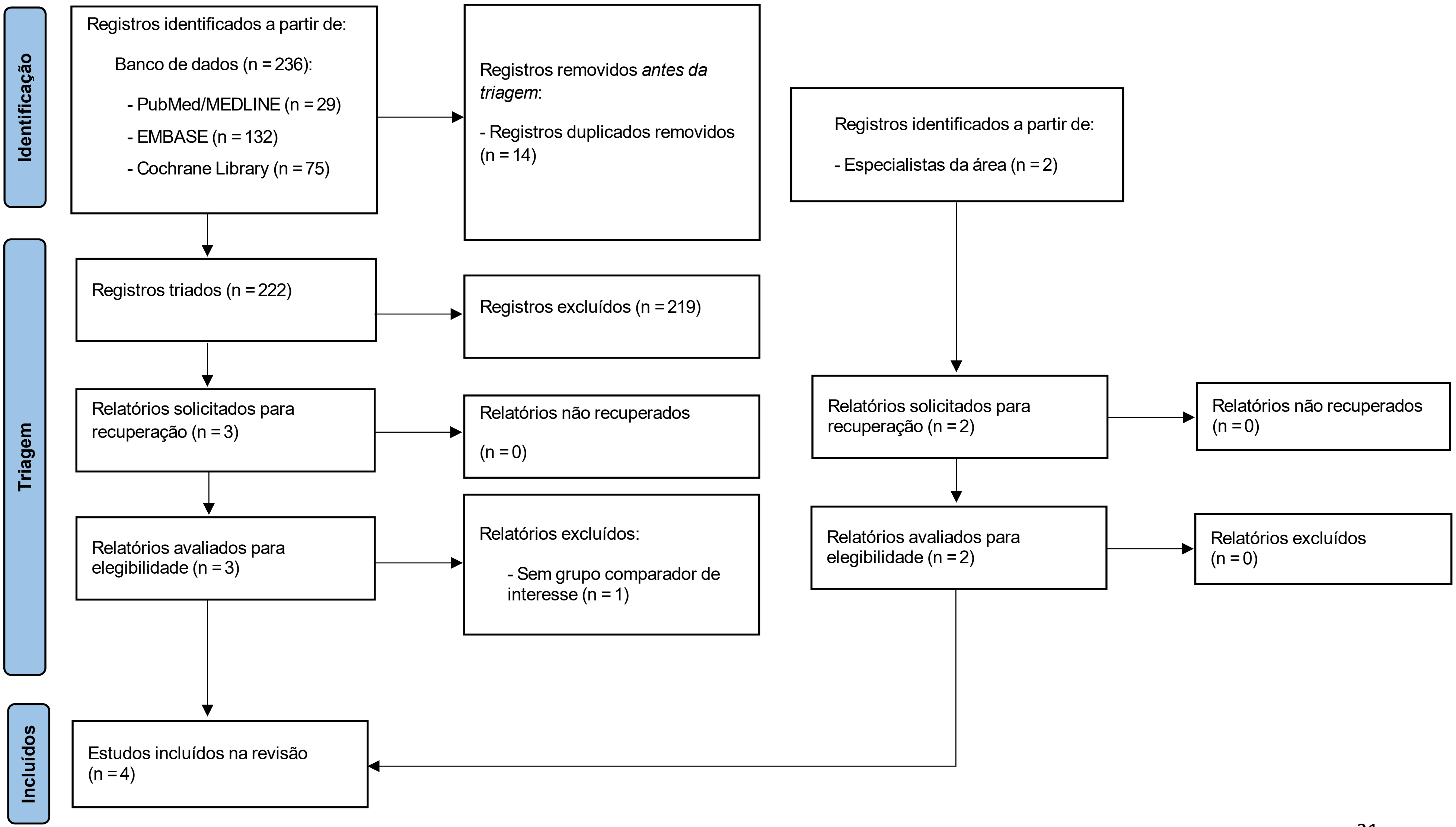

Supplement: Supplementary file 1 [file 0066-782x-abc-123-8-e20260423-suppl01-en.pdf]
