# Supplementary material for: Revisão Sistemática sobre a Segurança do Exercício Físico na Cardiomiopatia Hipertrófica: Recomendações da Sociedade Brasileira de Cardiologia
Source: Arq Bras Cardiol. 2026 Sep 8;123(8):e20260423. [Article in Portuguese] doi: 10.36660/abc.20260423 (PMC13671148; doi:10.36660/abc.20260423)
Supplement: Translation

# Translation: Systematic Review on the Safety of Physical Exercise in Hypertrophic Cardiomyopathy: Recommendations from the Brazilian Society of Cardiology

**DOI:** 10.36660/abc.20260423i

**Authors:** Ricardo Stein, Rodrigo Otávio Bougleux Alô, Luiz Eduardo Fonteles Ritt, Alexsandro Alves Fagundes, Anderson Donelli da Silveira, Artur Haddad Herdy, Clea Colombo, Cristiano Faria Pisani, Fabrício Braga, Frank Nunes, Luciana Sacilotto, Marconi Gomes Silva, Mauricio Milani, Mauricio Pimentel, Milena dos Santos Barros Campos, Nágela Simão Vinhosa Nunes, Pablo Marino Nascimento, Susimeire Buglia, José Ronyeryson dos Santos Evangelista, Filipe Ferrari, Leonardo Castro Luna

**Affiliations:** 1 Brazil Grupo de Pesquisa em Cardiologia do Exercício, Hospital de Clínicas de Porto Alegre, Porto Alegre, RS – Brazil; 2 Brazil Departamento de Clínica Médica, Universidade Federal do Rio Grande do Sul, Porto Alegre, RS – Brazil; 3 Brazil Programa de Pós-Graduação em Cardiologia e Ciências Cardiovasculares, Universidade Federal do Rio Grande do Sul, Porto Alegre, RS – Brazil; 4 Brazil Instituto Dante Pazzanese de Cardiologia, São Paulo, SP – Brazil; 5 Brazil Hospital Cardio Pulmonar, Salvador, BA – Brazil; 6 Brazil Escola Bahiana de Medicina e Saúde Pública – Pós-Graduação em Medicina e Saúde Humana, Salvador, BA – Brazil; 7 Brazil Hospital Ana Nery, Salvador, BA – Brazil; 8 Brazil Hospital de Clínicas de Porto Alegre, Porto Alegre, RS – Brazil; 9 Brazil Instituto de Cardiologia de Santa Catarina, São José, SC – Brazil; 10 Brazil Faculdade São Leopoldo Mandic, Campinas, SP – Brazil; 11 Brazil Instituto do Coração do Hospital das Clínicas da Faculdade de Medicina da Universidade de São Paulo, São Paulo, SP – Brazil; 12 Brazil Laboratório de Performance Humana, Rio de Janeiro, RJ – Brazil; 13 Brazil Universidade do Estado do Rio de Janeiro, Rio de Janeiro, RJ – Brazil; 14 Brazil Hospital César Leite, Manhuaçu, MG – Brazil; 15 Brazil Universidade Federal de Minas Gerais, Belo Horizonte, MG – Brazil; 16 Brazil Sportif - Clínica do Exercício e do Esporte, Belo Horizonte, MG – Brazil; 17 Brazil Fitcordis, Brasília, DF – Brazil; 18 Brazil Hospital São Lucas, Rede D’Or, Aracaju, SE – Brazil; 19 Brazil Complexo Hospitalar de Niterói, Niterói, RJ – Brazil; 20 Brazil Hospital Universitário Antônio Pedro, Niterói, RJ – Brazil; 21 Brazil Instituto Nacional de Cardiologia, Rio de Janeiro, RJ – Brazil; 22 Brazil Universidade do Grande Rio, Rio de Janeiro, RJ – Brazil

**Keywords:** Hypertrophic Cardiomyopathy, Exercise, Mortality, Systematic Review, Cardiac Arrhythmias

## Abstract

**Background:**

Although historically discouraged due to the risk of arrhythmias and sudden cardiac death (SCD), physical exercise (PE) may be safe and beneficial for individuals with hypertrophic cardiomyopathy (HCM).

**Objective:**

To evaluate the association between PE and the incidence of mortality and arrhythmic events in adults with HCM, stratifying the analysis according to different types and intensities of exercise.

**Methods:**

Systematic review of randomized clinical trials (RCTs) and observational studies identified in PubMed, Embase, and the Cochrane Library through December 2024. Studies including individuals exposed to different intensities of PE were eligible. Risk of bias was assessed using the RoB 2 and ROBINS-I tools, and certainty of evidence using GRADE. Recommendations were developed by an expert panel of the Brazilian Society of Cardiology.

**Results:**

Eight studies (four RCTs and four observational studies; 9,744 participants) were included. None reported an increased risk of mortality or severe arrhythmic events associated with PE, even at moderate or vigorous intensities. Syncope was rare. Additionally, a Korean observational study involving more than 7,000 participants (mean age: 59 years) demonstrated reduced all-cause mortality with higher physical activity intensity (dose–response curve). In low-risk athletes, competitive sports were not associated with severe adverse events. The quality of evidence ranged from moderate to very low.

**Conclusion:**

PE in HCM should be encouraged based on individualized assessment. In patients without high-risk criteria for SCD, benefits appear to outweigh risks. For higher-risk subgroups, recommendations are more cautious and based on expert consensus.

**Recommendation 1:** The Brazilian Society of Cardiology recommends the regular practice of physical exercise in patients with hypertrophic cardiomyopathy (HCM), emphasizing that therapeutic management should be individualized and established through shared decision-making between physician and patient. This recommendation aims to encourage patients with HCM to remain physically active, promoting overall health through regular and progressive physical exercise within an individualized prescription framework.

## STRONG RECOMMENDATION. MODERATE CERTAINTY OF EVIDENCE.

This recommendation is based on the absence of increased risk of sudden cardiac death associated with regular physical exercise in individuals with HCM, as demonstrated in clinical studies that included the following subgroups: symptomatic patients (New York Heart Association [NYHA] functional class II–III), patients with implantable cardioverter-defibrillators, and patients undergoing aerobic and/or resistance exercise at different intensities.

The individualized recommendation should consider each patient’s risk of sudden cardiac death, estimated based on clinical characteristics and through a validated prediction calculator (HCM Risk-SCD 2023),^1^ with periodic annual reassessments – or semiannual reassessments in patients with high-risk markers (see below) – and immediate reassessment upon the onset of new symptoms.

**Recommendation 2**: The Brazilian Society of Cardiology recommends the regular practice of physical exercise in patients with HCM who present markers of elevated risk for sudden cardiac death, aiming to improve overall health. Preferably, exercise should be performed in a supervised setting – such as in cardiovascular rehabilitation programs or facilities equipped with an automated external defibrillator. Finally, it is emphasized that therapeutic management must be individualized and defined through shared decision-making between the attending physician and the patient.

## WEAK RECOMMENDATION. VERY LOW CERTAINTY OF EVIDENCE.

High-risk markers include:

- symptoms such as dyspnea, syncope, or dizziness, especially when related to exertion;
- arrhythmias (such as nonsustained or sustained ventricular tachyarrhythmias) at rest or during exertion, as well as symptoms during exercise testing or cardiopulmonary testing;
- family history of sudden cardiac death in a first-degree relative younger than 50 years;
- septal thickness equal to or greater than 30 mm;
- extensive late gadolinium enhancement (> 15%) on cardiac magnetic resonance imaging;
- left ventricular outflow tract gradient ≥ 30 mmHg at rest or ≥ 50 mmHg with provocation (assessed by exercise echocardiography – provocation may be performed using Valsalva maneuver, exertion, post-exertion, or after squatting).

This approach should ideally include individualized prescription, clinical monitoring, education, psychological support, and management of comorbidities, aiming to ensure safety and improve quality of life. Individual preferences should be considered, and aerobic and resistance exercises should be combined whenever possible.

This recommendation is based on expert consensus, given the absence of specific studies involving the subgroup of patients with high-risk markers for sudden cardiac death.

**Recommendation 3**: The Brazilian Society of Cardiology does not contraindicate high-intensity exercise or participation in competitive sports for patients with HCM and, in carefully selected cases, recommends that this possibility be considered. For this purpose, high-risk markers must be absent, a prior evaluation must be conducted by a multidisciplinary team, and the final decision must be agreed upon between the attending physician and the patient (according to the criteria established below). This recommendation specifically applies to high-intensity exercise and to athletes in competitive settings, in which the central objective is performance and competition.

## WEAK RECOMMENDATION. LOW CERTAINTY OF EVIDENCE.

This recommendation is based on a small number of observational studies and on expert consensus. It is recommended that training and competition venues have a medical action plan in place to enhance safety during sports participation.

The decision should consider the following criteria:

1. Absence of high-risk markers for adverse events or sudden cardiac death (for example: history of ventricular arrhythmias, unexplained syncope, ventricular septal thickness ≥ 30 mm, or extensive late gadolinium enhancement on cardiac magnetic resonance imaging).
2. Prior multidisciplinary evaluation, preferably in a reference center for hereditary cardiomyopathies, including:
  Exercise testing (treadmill or cardiopulmonary) with analysis of blood pressure response;
  Prolonged electrocardiographic monitoring (e.g., 24–48-hour Holter);
  Cardiac magnetic resonance imaging with quantification of myocardial fibrosis.
3. Individualized risk stratification, preferably using the HCM Risk-SCD 2023 score,^1^ followed by shared decision-making with the patient.

## Introduction

Hypertrophic cardiomyopathy (HCM) is characterized by left ventricular (LV) hypertrophy not explained by hemodynamic overload or other secondary causes.^2,3^ The diagnosis is defined by a ventricular wall thickness ≥ 15 mm or, in the presence of a family history or a known genetic variant, ≥ 13 mm.^4^ HCM presents marked phenotypic heterogeneity, ranging from asymptomatic forms to severe presentations, including heart failure and sudden cardiac death (SCD).^5-7^ Population studies, such as the CARDIA study conducted in the United States, have estimated a prevalence of approximately 0.2% (1:500) in asymptomatic young adults,^8^ whereas more recent genetic analyses indicate a prevalence close to 0.5% (1:200).^9,10^

Historically, physical exercise (PE) has been discouraged in individuals with HCM due to the perceived risk of arrhythmias and SCD, especially among young athletes.^6,7^ However, recent evidence suggests that, when guided by individualized assessment and risk stratification, PE can be safe and beneficial for this population.^11-13^ In addition, studies indicate that high-intensity PE may be well tolerated and potentially advantageous in carefully selected patients.^12,13^ Nonetheless, uncertainties persist regarding the safety of different modalities and intensities of PE, particularly in higher-risk subgroups, reinforcing the need for individualized decision-making.

With the aim of grounding clinical practice in the best available scientific evidence, the Brazilian Society of Cardiology (SBC) developed this Medical Recommendation. This document analyzes the relationship between PE and the occurrence of severe arrhythmic events and mortality in patients with HCM, with stratified analysis according to exercise intensity and clinically relevant disease characteristics.

## Methods

To support this SBC Recommendation, a systematic review was conducted based on a research question structured according to the PICO/PECO format: “In adults with HCM, is the practice of PE at different types and intensities associated with a higher risk of mortality, SCD, or ventricular arrhythmias when compared with the absence of exercise or with lighter exercise intensities?”

The review protocol was previously registered in the International Prospective Register of Systematic Reviews (PROSPERO), under the number CRD420251005992.

It is important to emphasize that a rapid systematic review is a modality designed to support clinical decision-making using the best available evidence, maintaining scientific rigor while incorporating adaptations that allow for greater agility in execution. Its main characteristics include: restricting article language to English; searching gray literature only through the references of included studies, with support from field specialists; and using artificial intelligence to assist in screening and data extraction. This strategy has been widely adopted by medical societies and health institutions for the development of clinical recommendations.^14-16^

A detailed description of the methodology used in this rapid systematic review can be found in the Supplementary Material.

### Eligibility criteria

Studies were included if they met the following characteristics: (1) observational design or randomized clinical trials (RCTs); (2) inclusion of individuals with a clinical or genotypic diagnosis of HCM, regardless of sex; (3) investigation of PE, including aerobic training, resistance training, combined training (aerobic + resistance), or participation in specific sports modalities; (4) comparison between individuals who practiced and did not practice PE, or between different exercise intensities; and (5) assessment of at least one of the following outcomes: all-cause mortality, SCD, and ventricular arrhythmias (primary outcomes), as well as supraventricular arrhythmias, syncope, aborted cardiac arrest, and appropriate or inappropriate implantable cardioverter-defibrillator (ICD) shocks (secondary outcomes).

Studies that did not meet these criteria or did not evaluate relevant outcomes were excluded.

### Data sources and search strategies

Searches were conducted in three databases: PubMed/MEDLINE, Embase, and the Cochrane Library, from the earliest available date through December 18, 2024. Additionally, a manual search was performed in the references of retrieved documents, and articles of interest were requested from field specialists.

### Study selection

Study selection, extraction of key characteristics, and assessment of methodological quality were performed independently by two researchers. Any disagreements were resolved by consensus.

### Data extraction

Two researchers extracted predefined data into an electronic spreadsheet (Excel), including publication information, clinical and demographic characteristics of participants, and details of the interventions (type of exercise, intensity, frequency, duration, and supervision), as well as the results for the outcomes of interest.

### Quality assessment and risk of bias

Two researchers independently assessed the risk of bias using the Risk of Bias 2 (RoB 2)^17^ and Risk Of Bias In Non-randomized Studies – of Interventions (ROBINS-I)^18^ tools. Discrepancies were resolved by consensus. Certainty of evidence and strength of recommendations were evaluated according to the Grading of Recommendations, Assessment, Development and Evaluations (GRADE)^19^ framework.

### Data analysis

For each outcome, effect measures reported in the original studies were extracted, including relative risk, odds ratio, hazard ratio, mean differences, or absolute frequencies, as applicable. Study eligibility for each synthesis was determined based on clinical and methodological compatibility regarding population, type/intensity of exercise, comparators, and evaluated outcomes. When necessary, data were reorganized into standardized tables, harmonizing available information to facilitate comparison across studies.

Due to clinical and methodological heterogeneity among study designs, interventions, follow-up durations, and outcome definitions, results were synthesized narratively and presented in descriptive tables. Potential sources of heterogeneity were explored qualitatively, considering study design, exercise intensity, participant risk profile, and the context of sports participation. The robustness of conclusions was assessed based on the consistency of findings across studies, interpretation of risk of bias, and precision of estimates.

### Clinical recommendation and conflicts of interest

The clinical recommendation was established by consensus during a meeting of an SBC Recommendation Panel, composed of practicing cardiology specialists affiliated with the society. The project was supervised and funded by the SBC, while the development of the systematic review and the recommendation-formulation process were conducted by an independent team of methodologists (LCL, FF, JRES). This team declared no conflicts of interest related to the review, and the medical specialists on the panel did not receive honoraria or financial support.

## Results

### Study selection

Two independent searches were conducted: one for RCTs and another for observational studies, identifying 219 and 236 publications, respectively. After removing duplicates, eight original studies were included – four RCTs^11-13,20^ and four observational studies.^21-24^ The selection flowcharts (Figures 1S and 2S), search strategies (Tables 1S and 2S), and the list of potentially eligible but excluded studies, with corresponding justifications (Table 3S), are available in the Supplementary Material.

### Study characteristics

The eight included studies encompassed 9,744 participants, whose demographic characteristics are summarized in Table 1 (detailed data in Table 4S). The inclusion and exclusion criteria for the studies are presented in Table 5S. The observational study by Martinez et al.^24^ did not include exclusively individuals with HCM; therefore, some demographic and clinical variables of interest were not available separately for this subgroup.

**Table 1:** – Characteristics of the studies included

Study	Country	Design	Year	Sample (n)	Mean age (years)	Men (%)	Maximum mean LV wall thickness (mm)
Basu et al.^13^	United Kingdom	RCT	2025	80	46	84	Vigorous PE: 16 Usual care: 16
Gudmundsdottir et al.^20^	Denmark	RCT	2024	59	58	73	Moderate PE: 23 Usual care: 21
MacNamara et al.^12^	United States	RCT	2023	15	48	67	Moderate PE: 23 Vigorous PE: 24
Saberi et al.^11^	United States	RCT	2017	136	50	58	Moderate PE: 21 Usual care: 21
Kwon et al.^22^	South Korea	Retrospective cohort	2020	7,666	60	70	NR
Lampert et al.^21^	United States	Prospective cohort	2023	1,660	39	60	Moderately active: 21.4 Vigorously active: 20 Sedentary: 21.9
Martinez et al.^24^	United States	Retrospective cohort	2023	40	NR	NR	NR
Pelliccia et al.^23^	Italy	Retrospective cohort	2020	88	31 (median)	92	Trained athletes: 17 Untrained athletes: 17.3
**Study**	**ICD (%)**	**Obstructive (%)**		**LVEF(%)**	**Late enhancement**		**Symptoms**
Basu et al.^13^	Vigorous PE: 15 Usual care: 7.5	NR		65	NR		Participants in NYHA functional class II were considered eligible
Gudmundsdottir et al.^20^	Moderate PE: 24 Usual care: 17	0		58.5	NR		NYHA functional class II-III: Moderate PE: 86% Usual care: 70%
MacNamara et al.^12^	Moderate PE: 63 Vigorous PE: 0	Moderate PE 25^≠^ Vigorous PE: 38^≠^		65.5	NR		NR
Saberi et al.^11^	Moderate PE: 37.3 Usual care: 30.4	Moderate PE: 16.4^¥^ Usual care: 17.4^¥^		70.7	% LV mass: Moderate PE: 3.1 Usual care: 2.9		NYHA functional class II-III: Moderate PE: 34.3% Usual care: 33.3%
Kwon et al.^22^	NR	NR			NR		NR
Lampert et al.^21^	Moderately active: 41.4 Vigorously active: 40.6 Sedentary: 49.8	Moderately active: 18.8^¥^ Vigorously active:11.9^¥^ Sedentary: 21.8^¥^		66	Mild/moderate (< 15%): Sedentary: 16.2% Moderately active: 18.3% Vigorously active: 17.6% Extensive (> 15%): Sedentary: 6.5% Moderately active: 6.2% Vigorously active: 6.9%		NYHA functional class II: 24% (positive phenotype)^†^
Martinez et al.^24^	NR	NR		NR	NR		NR
Pelliccia et al.^23^	2/88 (2.3)	NR		65	Trained athletes: 22% Untrained athletes: 39%		Trained athletes: 22%* Untrained athletes: 25%*

ICD: implantable cardioverter defibrillator; RCT: randomized clinical trial; LV: left ventricular; NR: Not reported; NYHA: New York Heart Association; LVEF: left ventricular ejection fraction. ^≠^ Provocable left ventricular outflow tract obstruction. ^¥^ Resting left ventricular outflow tract obstruction. ^†^ Refers to participants with phenotypic hypertrophic cardiomyopathy (n = 1,534), of whom 24% presented exertional dyspnea (NYHA functional class II). * Values calculated from the proportion of asymptomatic participants (100% – % asymptomatic).

It is important to highlight that potentially higher-risk subgroups – such as individuals with ICDs, those with left ventricular outflow tract obstruction, patients with late gadolinium enhancement (myocardial fibrosis), and symptomatic individuals – were also represented among participants included in the RCTs evaluating moderate- or high-intensity exercise.

### Intervention characteristics

Intervention characteristics are summarized in Table 2 (detailed data in Table 6S). In the four RCTs,^11-13,20^ interventions lasted from 3^13,20^ to 5 months^12^ and included moderate continuous training, interval training (moderate or vigorous), and, in some cases, muscle strengthening. Sessions ranged from 20 to 60 minutes, with frequencies of 3 to 7 times per week and intensities between 60% and 95% of maximum or reserve heart rate. One study was fully in-person and supervised,^20^ others combined in-person and home-based sessions,^12,13^ and another used exclusively remote training.^11^

**Table 2:** – Interventions of the studies included

Study	Intervention	Intensity	Volume	Frequency	Duration	Comparator	Main results
Basu et al.^13^^¥^	Vigorous PE	70% of HR reserve gradually increasing to 85%	2 sessions (1 h/week) + 1 h of weekly home-based PE	3x/week	12 weeks	Usual care	There was no significant difference in the composite cardiovascular outcome^†^ between the groups
Gudmundsdottir et al.^20^^¥^	Moderate PE	60% of maximal work capacity (Borg scale 12–14)	10 minutes of warm-up, 30 min of interval training, 20 min of muscle strengthening	3x/week	12 weeks	Usual care	No major adverse events# were observed in either group
MacNamara et al.^12^^¥^	Moderate PE; moderate + progressively vigorous PE	Moderate: 1-20 bpm lower than ventilatory threshold Vigorous: 4×4 min at 90-95% of peak HR, with 3 min at ~70% of peak HR	Moderate: ≤ 4 sessions /week (1×60 min + 3×30 min) + 1 low-intensity session for recovery Vigorous: 14-15 sessions in 3 months: 10 min warm-up + 4×4 min interval training + 3 min active pause	~4x/week	5 months	No non-exercising control group	No severe arrhythmias or adverse cardiac events occurred
Saberi et al.^11^	Moderate PE	60% of HR reserve (Borg 11-14); progression to 70% of HR reserve in the 1^st^ month	20 min initially; progression to 60 min	Initially: ≥ 3x/week Progression: 4-7x/week	16 weeks	Usual care	No major adverse events^⁋^ occurred in either group
Kwon et al.^22^	Participants with the lowest, intermediate, or highest levels of PA	Classified into tertiles of the PA score^*^	Classified into tertiles of the MEE score, calculated using PA intensities of 3, 5, and 8 METs for light, moderate, and vigorous activities, respectively	NR	5.3 years	Low-intensity PA	Moderate to vigorous PA was associated with lower all-cause mortality and a trend toward lower cardiovascular mortality
Lampert et al.^21^	Vigorous or moderate PE	Moderate: 4.0-5.9 METs Vigorous: ≥ 6.0 METs	Moderate: ≥ 60 h/year in activities of 4 to < 6 METs; Vigorous: ≥ 60 h/year in activities ≥ 6 METs	NR	38 months	Sedentary	Vigorous PE did not increase deaths or serious arrhythmias compared with moderate PE or sedentary behavior
Martinez et al.^24^	Return to sports	NR (presumably high, given the competitive level)	NR (presumably high, given the competitive level)	NR	7 years	-	Return to sports participation is feasible and is associated with low rates of adverse events, even in elite athletes
Pelliccia et al.^23^	Maintenance of high-intensity training	High-intensity training	Trained group: ≥ 3 sessions/week, ≥ 2 h per session, totaling 6–14 h/week Untrained group: < 6 h/week (sedentary or occasional activity)	≥ 3x/week	7 years	Sedentary or occasional leisure activity < 6 h/week	In low-risk athletes, continuing to compete did not increase cardiac events or lead to clinical deterioration

PA: physical activity; PE: physical exercise; HR: heart rate; MEE: minimum energy expenditure, calculated in MET-min/week based on weekly frequency and the minimum duration of physical activities (20 min for vigorous and 30 min for moderate and light activities); METs: metabolic equivalents; NR: Not reported. * Calculated as a weighted daily average in METs/day: (8a + 5b + 3c)/7, where a, b, and c represent the days with vigorous, moderate, and light activity, respectively. ^¥^ Included supervised interventions. ^#^ Death, aborted cardiac arrest, sustained ventricular tachycardia, implantable cardioverter-defibrillator shock, or hospitalizations. ^⁋^ Death, aborted cardiac arrest, appropriate implantable cardioverter-defibrillator shocks, or sustained ventricular tachycardia. ^†^ Death, cardiac arrest, appropriate/inappropriate implantable cardioverter-defibrillator therapy, exercise-induced syncope, sustained ventricular tachycardia, nonsustained ventricular tachycardia, atrial arrhythmias lasting ≥ 30 seconds.

Among the observational studies, two stratified individuals from the general HCM population into three different levels of physical activity based on metabolic equivalents (METs).^21,22^ The other two observational studies focused on evaluating athletes with HCM: Pelliccia et al.^2 3^ assessed athletes who maintained or discontinued structured high-intensity training (≥ 6 h/week), whereas Martinez et al.^24^ followed semi-professional and professional athletes who either returned or did not return to intense exercise after shared decision-making.

Information on registration, financial support, and conflicts of interest for the included studies is presented in Table 7S. The PRISMA checklist for this document is provided in Table 8S.

### Evaluated outcomes

#### Mortality

None of the RCTs reported deaths related to the exercise programs, regardless of intensity.^11-13,20^ Similarly, the observational studies did not identify an increased risk of mortality associated with regular PE.^21-24^

In an observational study including 7,666 individuals (mean age 59 years), total and cardiovascular mortality progressively decreased with increasingly higher levels of self-reported physical activity, demonstrating a dose–response relationship.^22^ In another study conducted in younger individuals (mean age 39 years), vigorous exercise did not increase the risk of death or severe arrhythmias compared with groups classified as moderate exercise or sedentary.^21^

In the two cohorts composed exclusively of athletes with HCM,^23,24^ remaining in competition for up to seven years was not associated with a higher risk of sudden death or clinical deterioration, and the clinical events observed were not related to PE practice.

#### Arrhythmias, ICD shocks, and syncope

Overall, the studies did not show (when reported) an increase in sustained ventricular arrhythmias, ICD shocks, or syncope associated with PE. Episodes of nonsustained ventricular tachycardia were reported in both groups (intervention and control), with no significant differences.^12,13^ A single case of sustained ventricular tachycardia, without complications, occurred in the control group of one RCT.^13^ Martinez et al.^24^ reported, among athletes with HCM who returned to sports, one isolated event of cardiac arrest not related to exertion and one case of non-arrhythmic exertional syncope, with return to sports and no new episodes over 52 months.^24^

Detailed information on these outcomes for each study is presented in Table 9S.

#### Risk of bias

According to the RoB 2 tool, two RCTs were classified as having high risk of bias, one as having moderate risk, and one as having low risk (Figure 1). Among the observational studies evaluated with ROBINS-I, two showed high risk of bias, one moderate risk, and one low risk (Figure 2).

**Figure 1:** – RoB 2 of the randomized clinical trials. D1 Randomization; D2 Deviations from intended interventions; D3: Missing data; outcome; D4: Measurement; D5: Selection of reported results.

**Figure 2:** – ROBINS-I of the observational studies. (*) There was no control for confounders: high risk of bias; NI: not informed. D1: Confounding; D2: Classification of interventions; D3: Selection of participants; D4: Deviations from interventions; D5: Missing data; D6: Outcome measurement; D7: Reporting bias.

## Discussion

This systematic review supports the SBC recommendations regarding the safety of PE in individuals with HCM. Based on the findings, the potential risks of exercise are discussed across three groups: individuals with HCM from the general population, those with high-risk markers for SCD, and semi-professional or professional athletes.

### Historical and current context

Until recently, guidelines discouraged PE in individuals with HCM, mainly due to concerns about SCD in young athletes. The Bethesda Conferences,^25,26^ the European Society of Cardiology (2005),^27^ and the American Heart Association/American College of Cardiology (2015)^28^ adopted a restrictive stance regarding recreational or competitive sports of moderate to vigorous intensity in people with HCM.

Such restrictions may contribute to increased sedentary behavior (defined as waking activities with energy expenditure ≤ 1.5 METs, typically performed sitting, reclining, or lying down), physical inactivity (not meeting minimum physical activity recommendations for health), obesity, anxiety, and reduced quality of life.^29,30^ Resistance to prescribing exercise in this context persists today, partly due to the emotional impact of SCD cases and also due to limited awareness of current evidence. However, more recent guidelines from the American Heart Association/American College of Cardiology (2025)^31^ indicate that athletes with HCM – including carriers of pathogenic genetic variants and elite athletes – may be eligible to participate in vigorous exercise and competitive sports. This clearance, however, requires specialized evaluation and shared decision-making. Available data indicate that there is no significant increase in the risk of serious cardiac events.

### Consequences of exercise restriction and physical inactivity in patients with hypertrophic cardiomyopathy

In patients with HCM, particularly when formal contraindications to PE are present, the benefits associated with regular exercise for cardiovascular, metabolic, musculoskeletal, and mental health may be lost. In addition, reducing or discontinuing exercise may promote the development or worsening of metabolic alterations and increase individual cardiovascular risk, similar to what is observed in the general population.^32-38^

A qualitative study external to this systematic review suggests that active adults or athletes with HCM may experience difficulty adapting to sports restrictions, especially when there is a competitive history, strong athletic identity, or a substantial reduction in time dedicated to exercise.^39^ Considering its methodological limitations, these data may be used to contextualize patient values and preferences. Therefore, although they do not support strict clinical recommendations, these aspects reinforce the importance of cardiologists considering psychosocial factors, providing appropriate emotional support, and incorporating patient preferences during the shared decision-making process.

### Evidence from randomized clinical trials

Despite limitations such as short follow-up, small sample sizes, and the predominance of studies conducted in high-income countries,^11-13,20^ the available RCTs did not report significant adverse events, even in high-intensity protocols. In total, the studies followed approximately 80 patient-years without the occurrence of relevant adverse events, suggesting that high-intensity exercise may be safe in patients with HCM.

All four RCTs also observed improvements in functional capacity, regardless of the intensity or duration of the protocol. In a multicenter trial with 136 patients, peak oxygen consumption (VO_2_ peak) increased by an average of 1.27 mL·Kg^-^^1^·min^-^^1^ after 16 weeks of moderate exercise,^11^ whereas Basu et al.^13^ reported an average gain of 4.1 mL·Kg^-^^1^·min^-^^1^ with high-intensity exercise over 12 weeks. Part of these gains, however, was lost after six months, highlighting the importance of continued training.

These safety characteristics and cardiometabolic benefits contributed to Recommendations 1 and 2.

### Evidence from observational studies

This review included four observational studies: two involving athletes with HCM^23,24^ and two involving individuals with HCM from the general population.^21,22^ Although observational studies generally provide low-quality evidence (GRADE), they are useful for assessing the safety of PE over longer follow-up periods and its impact on mortality, given their larger sample sizes.

In studies involving athletes, returning to high-performance sports after specialized evaluation and shared decision-making was considered safe. Martinez et al.^24^ reported a low incidence of events among 40 athletes with HCM followed by a multidisciplinary team, with no deaths occurring during an average follow-up of seven years. In another retrospective study, 27 of 88 athletes with HCM continued training without adverse events and with clinical stability.^23^ These findings indicate that, in well-evaluated athletes, high-performance sports participation may be feasible and safe. However, the strength of these recommendations is limited by the retrospective nature and modest sample sizes. These findings were incorporated into Recommendation 3 of this document.

In studies conducted in the general HCM population, the results were also favorable. In a cohort of more than 7,000 individuals (mean age 59 years), a progressive reduction in mortality was observed with increasing intensity of physical activity (dose–response curve).^22^ Because this study was assessed as having low risk of bias, this finding resulted in upgrading the certainty of evidence from “Low” to “Moderate” regarding the benefit of exercise on mortality.

Consistently, another study with more than 1,600 participants (mean age 39 years) demonstrated that vigorous PE – including in competitive-level athletes (259 participants) – was not associated with an increased risk of adverse events when compared with lower-intensity exercise.^21^ These safety and mortality data contributed to Recommendations 1 and 2.

### Observational evidence not included in this review

Two observational studies not included in this review due to eligibility criteria – but which provide relevant information on the topic – are described below.

Basu et al.^40^ conducted a study with 53 athletes, all diagnosed with HCM described as morphologically mild and low-risk for SCD. Over 4.5 years of follow-up, no cases of sudden death, sustained ventricular tachycardia, or syncope associated with continued competitive sports participation were identified. The reported rate of asymptomatic arrhythmias was 2.1% per year, with minimal clinical impact.^40^

In another observational study with nearly nine years of follow-up, Desai et al.^41^ reported that asymptomatic or minimally symptomatic individuals with HCM who achieved >100% of the predicted METs on exercise testing had very low event rates, whereas those with lower functional capacity (<85%) had significantly higher risk.^41^ This finding reinforces the relevance of functional capacity not only as an efficacy outcome but also as a prognostic marker and a tool for risk stratification.

### Subgroups with specific characteristics

It is noteworthy that individuals with ICDs, symptomatic patients (NYHA functional class II–III), those with left ventricular outflow tract obstruction (at rest or provocable), and patients with mild myocardial fibrosis (<15%) were included in the studies. Extensive late gadolinium enhancement (>15%) was present in 6% to 7% of participants in one of the studies.^21^Therefore, the favorable recommendation for PE applies to most low-risk patients, including those with these characteristics, while the approach should be individualized for higher-risk cases.

### Late gadolinium enhancement on cardiac magnetic resonance

Late gadolinium enhancement is an important prognostic marker in HCM, associated with a higher risk of ventricular arrhythmias and SCD, especially when it exceeds 15% of LV mass – this threshold is adopted in this document as a criterion for high SCD risk.

Although there is no consensus on the best quantification technique, the presence of late gadolinium enhancement can assist in risk stratification, particularly in cases with ambiguous ICD indications, and in differentiating HCM from other causes of ventricular hypertrophy.^42,43^ However, a direct relationship between late gadolinium enhancement and PE has not yet been established by specific and conclusive studies.

### Patients with high-risk markers for sudden cardiac death

One of the greatest challenges in this document was addressing patients with HCM who have high-risk markers for SCD. There is no specific evidence regarding the safety of PE in this subgroup. Therefore, the SBC recommendation – based on consensus and caution – is favorable to PE, provided it is conditioned on individualized evaluation and shared decision-making, acknowledging the very low certainty of the available evidence.

### Limitations and future perspectives

This document presents important limitations. The first concerns the difficulty of conducting methodologically robust studies with long-term follow-up in low-prevalence diseases, which restricts the extrapolation of results to the general HCM population.

The second limitation relates to the heterogeneity of the interventions evaluated, both in terms of modality and intensity, as well as the clinical diversity of participants. This variability compromises the robustness of the conclusions and requires cautious interpretation.

Young adults (20-40 years), a subgroup traditionally at higher risk for SCD, were evaluated only in small observational studies, reducing the confidence of recommendations for this profile. This caution is reinforced by external epidemiological data showing that cardiovascular causes were the main category of sudden death in young adults aged 18–45 years, including some cases of HCM identified on autopsy.^44^

Additionally, applicability to the Brazilian context may be limited by factors such as restricted access to specialists and scarcity of cardiac rehabilitation services. Moreover, within the scope of this Recommendation, no studies conducted with Brazilian patients diagnosed with HCM are known.

Given these limitations, new studies – including prospective registries and RCTs with longer follow-up – are needed to strengthen the evidence base and, in the future, revise the recommendations presented here.

## Conclusion

PE and sports participation in the setting of HCM should be encouraged based on individualized evaluation, considering factors such as clinical profile, cardiovascular risk, and patient preferences. In patients without high-risk criteria for SCD, the benefits of exercise — including improvements in functional capacity, quality of life, and potential cardioprotective effects — appear to outweigh the risks.

However, for higher-risk subgroups, such as those with severe arrhythmias, a history of cardiac arrest, or significant ventricular dysfunction, recommendations should be more cautious. In these cases, decisions should be made through multidisciplinary evaluation and a shared decision-making process with the patient, following a careful analysis of the risk-benefit balance. Additional studies are needed to better stratify risk and define personalized exercise protocols for this population.

## *Supplemental Materials

Supplementary material For additional information, please click here https://abccardiol.org/supplementary-material/2026/12308/2026-0423_AO_Material-suplementar.pdf
